# Harnessing Contact-Quenched, Profluorescent Chemical Probes for Sensitive Determination and High-Throughput Measurements of Enzyme Activity

**DOI:** 10.1021/acsomega.5c06578

**Published:** 2025-11-22

**Authors:** Chien-Hui Huang, Chia-Yen Dai, Su-Hung Wang, Scott Severance, Chi-Ching Hwang, Yu-Chen Liu, Bao-Lin Yeh, Yung-Chieh Weng, Siao-Lei Yu, Hsing-Tao Kuo, Li-Fang Wang, Jeh-Jeng Wang, Tzu-Pin Wang

**Affiliations:** † Department of Medicinal and Applied Chemistry, 38023 Kaohsiung Medical University, Kaohsiung 80708, Taiwan; ‡ School of Medicine and Drug Development and Value Creation Research Centre, Kaohsiung Medical University, Kaohsiung 80708, Taiwan; § Department of Internal Medicine, Kaohsiung Medical University Hospital, Kaohsiung Medical University, Kaohsiung 80708, Taiwan; ∥ Division of Hepato-Gastroenterology, Department of Internal Medicine, 38018 Chi Mei Hospital, Tainan 72263 Taiwan; ⊥ Department of Molecular and Cellular Sciences, 418678 Liberty University College of Osteopathic Medicine, Lynchburg, Virginia 24515, United States; # Department of Biochemistry, 38023 Kaohsiung Medical University, Kaohsiung 80708, Taiwan; ∇ Department of Medical Research, Kaohsiung Medical University Hospital, Kaohsiung Medical University, Kaohsiung 80708 Taiwan

## Abstract

Dual-labeled, profluorescent chemical probes have been developed to quantify and visualize a specific enzyme’s activity in complex biological media. The intact chemical probes often exhibit minimal fluorescence due to fluorescence quenching, but their intrinsic fluorescence can be released by a favorable enzyme-catalyzed reaction. Contact quenching represents one of several fluorescence quenching mechanisms. However, it is seldom intentionally implemented in the synthesis of dual-labeled, profluorescent chemical probes, because the structure of a contact quenching construct requires the formation of a ground-state fluorophore-quencher/-fluorophore complex. The ability of such dual-labeled molecular probes to act as intramolecular dimers cannot be predicted. We previously revealed that a *mono exo*-bicyclo­[6.1.0]­nonyne (*exo*-BCN)-derivatized cystamine framework was critical to synthesizing sensitive, dual-labeled, profluorescent chemical probes capable of contact quenching. Here, we exploited the nonsymmetrical *mono*-*exo*-BCN-cystamine backbone by sequentially coupling it with two different carboxyfluorescein (FAM) derivatives and subsequently synthesizing four nonsymmetrical *bis*-FAM chemical probes. The fluorescence turn-on properties of the *bis*-FAM chemical probe with the lowest background FAM fluorescence were characterized by kinetic studies. Moreover, the release of two FAM equivalents from the fluorescence turn-on chemical probe during reactions was utilized to develop sensitive assays for measuring the activity and inhibition of two serum biomarkers, butyrylcholinesterase (BChE) and paraoxonase 1 (PON1) lactonase. We also developed an efficient, high-throughput assay for detecting BChE activity based on the chemical probe. Finally, the fluorescence assays successfully quantified the activities of BChE and PON1 lactonase in human serum.

## Graphical abstract

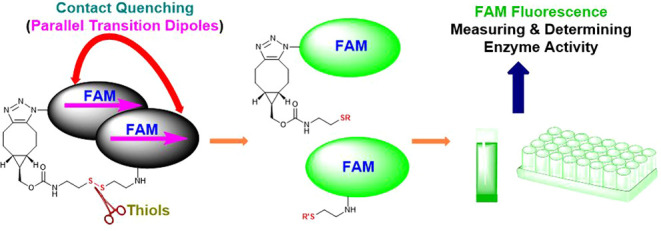

## Introduction

Fluorophore-labeled probes are used in a wide variety of chemical and biochemical assays. Many of these assays exploit profluorescent probes in biochemical processes to increase the fluorescence signal in order to facilitate the detection and quantification of intrinsic activities in biological systems.^1,2^ Profluorescent probes are typically synthesized by incorporating one or two fluorophores into the fluorescence-quenched constructs.^1,2^ Dual-labeled, profluorescent probes can contain fluorophore-quencher or fluorophore–fluorophore pairs and have background fluorescence intensity intricately controlled by the fluorophore-quencher or fluorophore–fluorophore distance. Molecular beacons are prominent examples of dual-labeled profluorescent probes used in DNA/RNA analysis.^3,4^ Close contact of the reporter and quencher moieties in molecular beacons ensures effective fluorescence quenching contributed by contact quenching effect.^5,6^

Effective contact quenching requires the formation of an intramolecular, nonfluorescent ground-state dimer between the moiety of a reporter fluorophore and the moiety of an acceptor, which can be either a fluorophore or a quencher.^5,6^ The close contact of dye–dye-paired moieties in contact quenching constructs promotes strong, intramolecular, dipole–dipole coupling of the dye components and perturbs the absorbance of intramolecular ground-state, reporter-acceptor complexes.^4–11^ Therefore, the visible absorption spectrum of a fluorophore-acceptor pair in a contact quenching construct is noticeably different from the spectrum when the acceptor is too distant to interact with the fluorophore.^4–10^ In addition, after irradiation at an excitation wavelength, resonance dipole–dipole interactions of ground-state complexes in contact quenching constructs will nonradiatively transfer the energy to the surrounding environment primarily as heat, with only a limited amount of the energy released as fluorescence. Moreover, in effective contact quenching constructs, all of the fluorophores are quenched equally by the acceptor, regardless of whether or not the emission spectrum of a fluorophore overlaps the absorption spectrum of the acceptor.

The spectroscopic characteristics of contact quenching constructs is explained by exciton theory,^5,9,10,12,13^ which explains that the formation of an intramolecular ground-state complex between two dyes allows strong coupling between their transition dipoles, and results in the delocalization of excited electrons over the two dyes, the development of exciton absorption bands, and significant changes in the absorption spectrum. Furthermore, the two dyes can form H-aggregate dimers with parallel transition dipoles and generate a blue shift in the absorption spectrum and fluorescence quenching in contact quenching constructs. Fluorescence quenching of H-aggregate dimers in contact quenching constructs is attributed to electron transitions occurring from only the ground state to the exciton state with the highest energy. Excited electrons then experience a rapid, internal conversion and proceed to the exciton state with a lower energy, which prohibits radiative transitions to the ground state and results in a radiationless intersystem crossing process and fluorescence quenching.^10^ Moreover, exciton theory suggests that a dual-labeled, contact quenching-based, profluorescent probe affords the following advantages: reduces the constraints on acceptor dye selection, makes the use of the same fluorophore as the reporter and acceptor dye moieties in a construct feasible, releases two equiv of fluorophores from said contact quenching constructs, and, most importantly, affords the development of sensitive fluorescence assays.

The ability to incorporate two identical fluorescent moieties into dual-labeled, contact quenching-based, profluorescent probes has significant advantages over other profluorescent probes such as those based on the fluorescence quenching mechanisms of Förster resonance energy transfer (FRET), through-bond energy transfer (TBET), and photoinduced electron transfer (PeT).^14^ Specifically, dual-labeled, FRET-based, profluorescent probes require that the spectra of the excited-state fluorophore emission and ground-state quencher absorption overlap as much as possible and that the distance of fluorophore-quencher pairs are within a Förster distance, which is equal to the distance between a FRET pair that corresponds to 50% energy transfer efficiency.^14^ Additionally, no perturbation of visible spectra of fluorophores and quenchers is observed in dual-labeled, FRET-based, profluorescent probes. Typically, only one equivalent of a fluorophore can be released from FRET-based probes. Similarly, TBET- and PeT-based, profluorescent probes usually contain a single fluorophore moiety and, by design, release one equivalent of the respective fluorophore after fluorescence quenching is obliterated. Resultingly, dual-labeled, contact quenching-based, profluorescent probes have stronger fluorogenic properties and are excellent candidates for use in a variety of sensitive assays, including biomedical applications.

However, the synthesis of dual-labeled, contact quenching-based, profluorescent probes is not often easily achieved because, as indicated above, a contact quenching construct must form a ground-state fluorophore-acceptor complex, which is typically detected and appreciated only after the compound has been synthesized. We recently revealed that a *mono exo*-bicyclo­[6.1.0]­nonyne (*exo*-BCN)-derivatized cystamine framework was critical to synthesizing sensitive, dual-labeled, profluorescent chemical probes capable of contact quenching.^7,15,16^ Each of the fluorescence turn-on chemical probes displayed characteristic visible absorption changes contributed by the contact quenching mechanism. The results supported our assertion that effective contact quenching in the chemical probes was due to derivatives of ***exo-*** **BCN-4** (Scheme S1, SI) participating in strain-promoted azide–alkyne cycloaddition (SPAAC)^17^ and forming a unique tricyclic fused ring structure in the chemical probes, which affected the steric structure of the chemical probes and activated contact quenching.^7^

The current research garnered critical knowledge concerning effective, ***exo-*** **BCN-4**-promoted contact quenching in dual-labeled, profluorescent chemical probes; exploited the nonsymmetrical *mono*-*exo*-BCN-cystamine backbone of ***exo-*** **BCN-6** (Scheme S1) synthesized from ***exo-*** **BCN-4**; sequentially coupled ***exo-*** **BCN-6** with two different carboxyfluorescein (FAM) derivatives; and subsequently synthesized four nonsymmetrical *bis*-FAM fluorescence turn-on chemical probes (Scheme S2, SI). The *bis*-FAM chemical probe with the lowest background FAM fluorescence was designated **asym6–6FAM** (Figure 1A and Scheme S2), and its fluorescence turn-on properties were characterized by kinetic studies.

**Figure 1:**
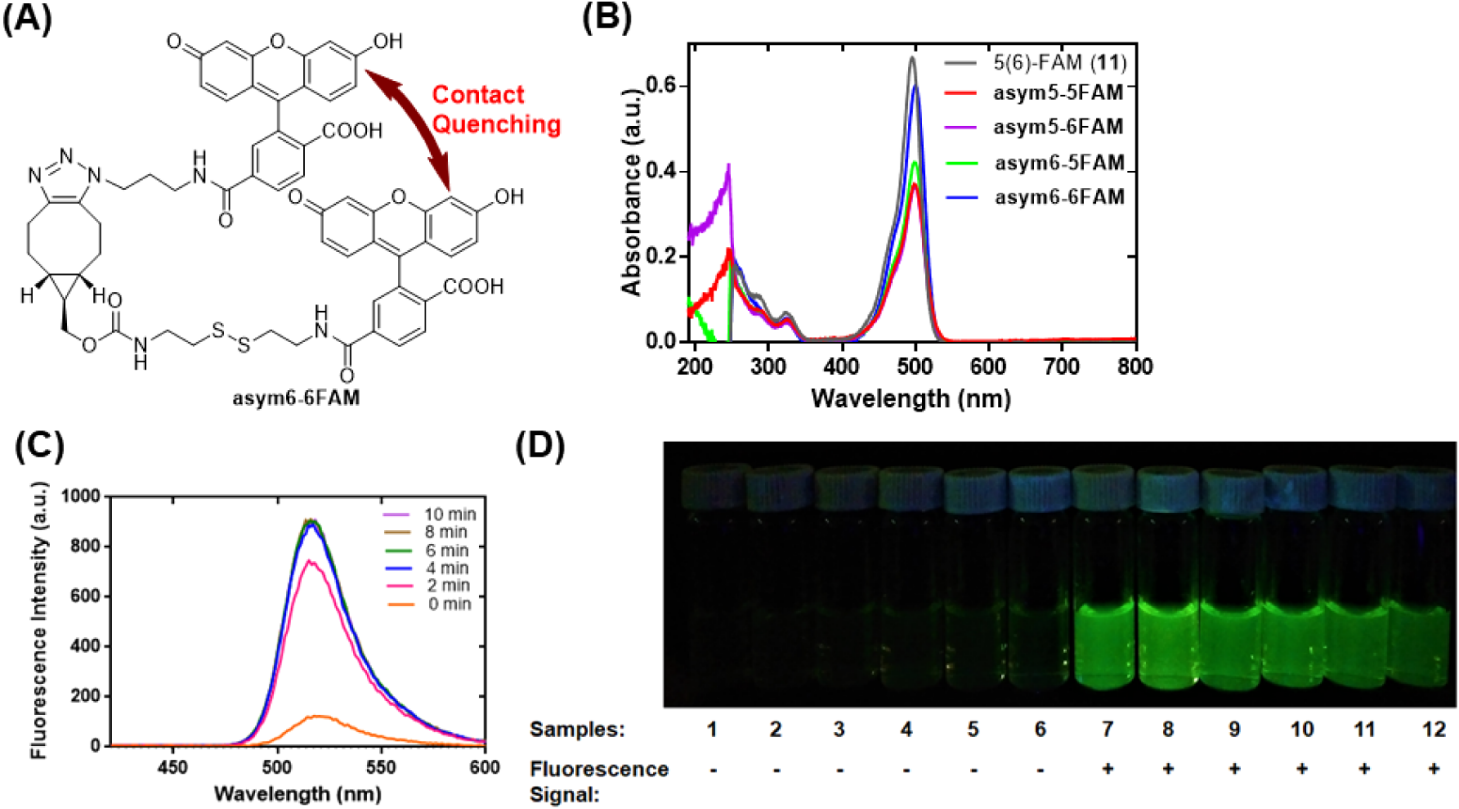
Contact quenching of fluorescence in the four nonsymmetrical *bis*-FAM chemical probes and release of the intrinsic fluorescence associated with the chemical probe **asym6–6FAM**, as determined by UV–vis absorption, fluorescence measurements, and a qualitative, fluorescent vial assay. (A) Structure of **asym6–6FAM**. (B) UV–vis spectra of the four nonsymmetrical *bis*-FAM chemical probes (5 μM each) and **11** (10 μM). (C) The time-dependent increase of the 6-FAM fluorescence in a reaction of **asym6–6FAM** (0.5 μM) with 2-aminoethanethiol (2-AET; 50 mM) in PB (100 mM, pH 7.4) in the presence of 1% DMSO at 25 °C. (D) The contact quenching of fluorescence and fluorescence emission of **asym6–6FAM** (2.5 μM) occurred in the presence of various reactants. Samples 2–12 included the reactants (50 mM) l-glutamate, glycine, l-serine, l-lysine, l-methionine, l-cysteine, glutathione (GSH), 2-mercaptoethanol (2-ThioEtOH), 2-AET, dl-dithiothreitol (DTT), and 1-butanethiol (nBuSH), respectively.

The ability of **asym6–6FAM** to release two FAM equivalents during appropriate reactions allowed us to develop sensitive assays for determining the activity and the inhibition of two serum biomarkers – butyrylcholinesterase (BChE) and paraoxonase 1 (PON1) lactonase. Both BChE and PON1 have been confirmed to be the major esterases in human plasma.^18^ The chemical probe **asym6–6FAM** was further employed to develop an efficient, high-throughput assay for quantifying the BChE activity. Finally, the innovative fluorescence assays successfully measured the activities of BChE and PON1 lactonase in human serum samples.

This report sheds light on the important structural components of nonsymmetrical, *mono*-*exo*-BCN-cystamine-based ***exo-*** **BCN-6** essential for effective contact quenching in dual-labeled, profluorescent chemical probes. The study demonstrated that ***exo-*** **BCN-6**-based fluorescence turn-on chemical probes can be employed to sensitively determine the activities of important biomarkers in biological systems. This information may prove to be essential to designing additional contact-quenching-dependent chemical probes and novel materials for applications requiring specific steric structures derived from ***exo-*** **BCN-6** involved in SPAAC reactions.

## Experimental Section

The detailed procedures for synthesis of the four chemical probes, kinetic characterization of **asym6–6FAM**, development of the **asym6–6FAM**-based activity assays of BChE and PON1 lactonase, and measurement of enzyme activity in human serum by fluorescence spectrometer-based or microplate-based, high-throughput assays are presented in the Supporting Information (SI). This study was conducted according to the guidelines of the Declaration of Helsinki and approved by the Ethics Committee of Kaohsiung Medical University Hospital [KMUHIRB-E­(I)-20200127]. The participants gave written informed consent.

## Results and Discussion

### Synthesis of Nonsymmetrical, *Mono-exo*-BCN-Based and *Bis*-FAM-Containing Fluorescence Turn-On Chemical Probes

Previous studies^7,15,16^ unambiguously demonstrated that the *exo-*BCN moiety in ***exo-*** **BCN-6** (Scheme S1) produced a unique tricyclic fused ring system during SPAAC reactions, affected the steric structure of the reaction products, and, most critically, bolstered effective contact quenching of fluorescence in constructs synthesized by coupling two different fluorescent molecules to two specific functionalities in the nonsymmetrical ***exo-*** **BCN-6**. The results motivated us to exploit the crucial structure of ***exo-*** **BCN-6** to develop advanced, contact quenching-based, fluorescence turn-on chemical probes.

In the current study, we synthesized four nonsymmetrical, *mono-exo*-BCN-based and *bis*-FAM-containing fluorescence turn-on chemical probes (Scheme S2). We employed distinct constitutional isomers of FAM derivatives from **11** (Scheme S3, SI) for synthesis of the FAM-derivatized chemical probes because recent research showed that a specific constitutional isomer of FAM in chemical probes had different effects on fluorescence quenching essential to sensitive measurements of chemical activity by fluorescence turn-on assays.^7,15^ Here the design of the chemical probe allowed us to acquire two homo-*bis*-FAM chemical probes by incorporating either two 5-carboxyfluorescein (5-FAM) or two 6-carboxyfluorescein (6-FAM) moieties into ***exo-*** **BCN-6**. Additionally, two hetero-*bis*-FAM chemical probes were systematically synthesized by coupling an NHS-ester of a specific constitutional isomer of FAM (**7** or **8**; Scheme S3)^15^ to ***exo-*** **BCN-6** to afford either ***exo-*** **BCN-9** or ***exo-*** **BCN-10** and by subsequently performing SPAAC with an azido derivative of the other constitutional isomer of FAM (**14a** or **14b**,^7^ Scheme S3). We expected only subtle differences in the spatial separation of two FAM components in the chemical probe structures. However, we believed that even a miniscule difference in distances between the two FAM moieties in the chemical probes would produce differential contact quenching effects on the FAM fluorescence and provide a profluorescent chemical probe with low background FAM fluorescence. Moreover, the identified chemical probe could release two equiv of the FAM fluorescence after the internal disulfide bond was cleaved, which would dismantle the intact structures of the chemical probe and abolish contact quenching effects in the construct. Consequently, fluorescence turn-on assays based on the *bis*-FAM-containing chemical probe would provide higher sensitivity than those capable of releasing only one equivalent of a specific fluorophore during reactions.

We succeeded in synthesizing the four chemical probes with satisfactory to good yields (51–79%) by employing SPAAC reactions between **14a**/**14b** and ***exo*** **-BCN-9**/***exo*** **-BCN-10** ^15^ (Scheme S2). The four chemical probes all displayed good properties of storage stability and interbatch repeatability after aliquoted, dried, and stored in −20 °C over two years (results not shown). In addition, we noticed that the ***exo*** **-BCN-10**-derived chemical probes, i.e., **asym6–5FAM** and **asym6–6FAM**, were obtained with lower yields (51–61%) than those (78–79%) of the chemical probes **asym5–5FAM** and **asym5–6FAM** synthesized from ***exo*** **-BCN-9**. Different yields for the chemical probes derived from ***exo*** **-BCN-9** or ***exo*** **-BCN-10** were consistent with information gleaned from a previous study reporting the synthesis of two chemical probes for quantifying PON1 lactonase activity.^15^ Fang et al. employed either ***exo*** **-BCN-9** or ***exo*** **-BCN-10** in a SPAAC reaction with an azido-derivatized rhodamine B and synthesized the two FAM-rhodamine B-paired chemical probes with differential yields. Specifically, the ***exo*** **-BCN-9**-derived chemical probe was synthesized with a yield of 95%, while the probe based on ***exo*** **-BCN-10** afforded a yield of 89%.^15^ As suggested before, this difference in yields between ***exo*** **-BCN-9-** and ***exo*** **-BCN-10**-based SPAAC reactions was likely caused by greater steric hindrance and molecular crowdedness in ***exo*** **-BCN-10** affecting the accessibility of the BCN moiety in the molecule for SPAAC reactions with azido counterparts and compromising the yield of the SPAAC products. Counterintuitively, the geometrical constraints of the 6-FAM moiety may allow the ***exo*** **-BCN-10**-dependent SPAAC reactions to synthesize chemical probes with fluorogenic properties that are more advantageous for developing sensitive enzyme assays, which will be elucidated in subsequent studies of the chemical probes.

### Spectroscopic Studies of the Four Nonsymmetrical *Bis*-FAM Chemical Probes

Contact fluorescence quenching in the four nonsymmetrical *bis*-FAM chemical probes was first characterized by UV–vis spectrophotometry (Figures 1B and S1A). We were initially intrigued, because the FAM absorbance in the UV–vis spectra of these four chemical probes was not significantly different from that of 5(6)-FAM (**11**). We expected the FAM moieties in the four nonsymmetrical *bis*-FAM chemical probes to exhibit a hypsochromic shift (blue shift) in their local maximum absorption relative to a monomeric FAM because the two FAM moieties in the molecules would form xanthene H-aggregates by maintaining a “side-by-side” orientation of their transition dipoles to perturb the UV–vis absorption and, more importantly, would result in quenching of the FAM fluorescence.^11^

The conundrum was explained by worked previously performed utilizing UV–vis absorption of several FAM-containing contact quenching probes used to determine the activity of elastase – a serine protease.^9,10^ The peptide probes used to determine elastase activity were synthesized by conjugating two fluorophores to two discrete functionalities in the peptide (designated NorFES) to obtain homo-*bis*-chromophoric or hetero-*bis*- chromophoric constructs. Interestingly, similar to the results displayed in Figure 1B, the UV–vis spectra of the profluorescent probes FAM-NorFES-FAM and 6-FAM-NorFES-carboxyrhodamine X all displayed insignificant changes in the FAM absorbance before and after hydrolysis of the probe structures. Nevertheless, the two intact NorFES-based probes quenched the FAM fluorescence. The FAM-NorFES-FAM probe quenched 55% of the FAM fluorescence, and the 6-FAM-NorFES-carboxyrhodamine X probe quenched 41% of the 6-FAM fluorescence.^9,10^ In contrast, the *bis*-rhodamine-based NorFES probes were far more effective at quenching rhodamine fluorescence (90–94% reduction). The fact that the formation constant for the FAM dimer in water (5 M^–1^) is approximately 3 orders of magnitude lower than those of other xanthene dyes such as rhodamine 6G (1695–6200 M^–1^) might explain the observed spectroscopic properties of the two FAM-containing probes built on NorFES.^11^ The lower affinity between FAM and the other xanthene fluorophore in the two NorFES-based probes led to reduced dipole–dipole interactions of the two xanthene fluorophores and resulted in less perturbation in the UV–vis absorption of the constructs. Nevertheless, the absence of significant alterations in the absorption spectra for the two NorFES-based probes still supported the existence of dipole–dipole interactions in intramolecular ground-state complexes and of sufficient contact quenching of fluorescence in the profluorescent probes.^9,10^ We wondered whether the same weak dipole–dipole interactions yet still significant contact quenching effects might also be inherent to the four nonsymmetrical *bis*-FAM chemical probes. We decided to further study the properties of fluorescence quenching of our chemical probes.

We are pleased to report that all four nonsymmetrical *bis*-FAM chemical probes are capable of quenching the FAM fluorescence while retaining the integral structures of the chemical probes (the 0 min spectra in Figures 1C and S1B–D). In addition, the swift, thiol-responsive, fluorescence turn-on characteristics of the four nonsymmetrical *bis*-FAM chemical probes were confirmed by measuring the time-dependent fluorescence in reactions between the chemical probes (0.5 μM) and 2-aminoethanethiol (2-AET, 50 mM) in phosphate buffer (PB; 100 mM, pH 7.4) (Figures 1C and S1B–D). Complete release of the FAM fluorescence from the four nonsymmetrical *bis*-FAM chemical probes due to the presence of 2-AET occurred within 4 min. Additionally, no change was detected in 6-FAM fluorescence levels when 6-FAM (1 μM) was incubated with 2-AET (50 mM) for 10 min (results not shown). Clearly, all four nonsymmetrical *bis*-FAM chemical probes had the desired properties of quenching the FAM fluorescence and of reemitting the FAM fluorescence in the presence of a thiol such as 2-AET.

Moreover, as demonstrated in Figures 1C and S1B–D, the efficiencies of contact quenching of the FAM fluorescence from the four nonsymmetrical *bis*-FAM chemical probes were noticeably different. Specifically, background fluorescence levels (ratio of fluorescence intensity of λ_max_ at time 0 relative to the maximum intensity of λ_max_ expressed in %; λ_max_ = 515 nm in this study) in the four nonsymmetrical *bis*-FAM chemical probes were 13% for **asym5–5FAM**, 17% for **asym5–6FAM**, 22% for **asym6–5FAM**, and 12% for **asym6–6FAM**. These values are equivalent to 87%, 83%, 78% and 88% quenching efficiency (calculated by subtracting % of the background fluorescence from 100% for each *bis*-FAM chemical probe) on the FAM fluorescence, respectively, for the four nonsymmetrical *bis*-FAM chemical probes (Table S1, SI). In addition, the background fluorescence levels in **asym5–5FAM** and **asym6–6FAM** were almost identical to the background fluorescence values of 14% at 505 nm and 13% at 525 nm, respectively, for the *mono*-*exo*-BCN-based chemical probes used to measure BChE or PON1 activity.^15,16^ We also measured the absolute quantum yield (Φ_*A*_) for **11** and the four chemical probes (Figure S2, SI), and then we determined the following values of quenching efficiencies for the four chemical probes: 92% for **asym5–5FAM**, 89% for **asym5–6FAM**, 86% for **asym6–5FAM**, and 95% for **asym6–6FAM** (Table S1). Therefore, values of quenching efficiencies for the four chemical probes obtained from fluorescence intensity at 515 nm and from Φ_*A*_ measurements were similar and comparable (Table S1).

The results from Table S1 and Figures 1C, S1B–D, and S2 strongly supported the assertion that the *mono*-*exo*-BCN-derived chemical probes such as **asym5–5FAM** and **asym6–6FAM** had more robust parallel dipole–dipole interactions and more significant contact quenching effects in their FAM moieties than those in the previously reported, FAM-containing, and NorFES-based probes. Since the chemical probe **asym6–6FAM** demonstrated the lowest background FAM fluorescence, highest quenching efficiency on the FAM fluorescence, and strongest intensity of the FAM fluorescence among the four nonsymmetrical *bis*-FAM chemical probes under the same concentration (0.5 μM; Table S1, Figures 1C, S1, and S2), we decided to further study **asym6–6FAM** using a qualitative, fluorescent vial assay. The results of this assay provided evidence for contact quenching of fluorescence in reactant-free **asym6–6FAM** and in reactions of **asym6–6FAM** with nonthiol reactants (Samples 1–6, Figure 1D). Moreover, the fluorogenic properties of **asym6–6FAM** were also revealed by the same vial assay when **asym6–6FAM** was reacted with various compounds bearing one or two thiol groups (Samples 7–12, Figure 1D). The favorable properties of contact quenching and turn-on of the 6-FAM fluorescence in **asym6–6FAM** inspired us to further study the chemical probe and explore its potential to sensitively measure the chemical activity.

### Characteristics of Reactions between **asym6–6FAM** and Thiols

We initiated kinetic studies on fluorogenic reactions of **asym6–6FAM** with thiols in order to evaluate the potential use of **asym6–6FAM** for measuring the activity of chemical reactions including catalysis by enzyme biomarkers. As shown in Figure 1C, **asym6–6FAM** promptly reacted with thiol 2-AET, and the contact quenching of the 6-FAM fluorescence in **asym6–6FAM** was completely obliterated within 4 min. The rapid reactivity of **asym6–6FAM** with thiols was further corroborated by the pseudo-first-order rate constants (*k*_1_) obtained in reactions of **asym6–6FAM** with 12 reactants (Figures S3A and S4, SI). All thiol-containing reactants – including GSH, thiocholine [one of the hydrolyzed products of *S*-butyrylthiocholine (BTCh) during BChE catalysis], and nBuSH [one of the hydrolyzed products of 5-thiobutyl butyrolactone (TBBL) during PON1 lactonase catalysis] – reacted with **asym6–6FAM** and increased levels of the 6-FAM fluorescence. Among all reactants, 2-AET obliterated the contact quenching effect in **asym6–6FAM** and released the 6-FAM fluorescence (*k*_1_ of 0.181 ± 0.004 min^–1^) most efficiently. A good reactivity of 2-AET with **asym6–6FAM** was similarly reported for reactions of 2-AET with two other fluorescent chemical probes developed by us.^7,15^ Thiocholine also reacted quickly with **asym6–6FAM** (*k*_1_ of 0.117 ± 0.003 min^–1^). The apparently biphasic progress curve for the thiocholine-**asym6–6FAM** reaction suggested that a simple pseudo-first-order mechanism could not account for the reaction, even in the presence of thiocholine concentration (5 mM) almost 4 orders of magnitude higher than **asym6–6FAM** (0.15 μM) in the reaction (Figure S4B). In addition, the moderate reactivity of GSH with **asym6–6FAM** (*k*_1_ of 0.033 ± 0.001 min^–1^) mirrored results from the study on the time-dependent decrease of fluorescence quenching and the subsequent increase of the 6-FAM fluorescence in the reaction of GSH (5 mM) with **asym6–6FAM** (0.5 μM) in PB (Figures S4E and S5, SI). The reaction took approximately 72 min to attain signal saturation of the 6-FAM fluorescence. Finally, we exploited the swift reactivity of 2-AET with **asym6–6FAM** and further characterized the kinetic properties of the 2-AET-**asym6–6FAM** reaction in order to ascertain critical information concerning the general reaction mechanism of **asym6–6FAM** with thiols.

We elucidated the mechanism of **asym6–6FAM** reactions with thiols using kinetic studies of 2-AET-**asym6–6FAM** reactions with varying 2-AET concentrations under differing pH values and containing metal ions. We first determined the *k*_1_ values of the reactions of **asym6–6FAM** under different 2-AET concentrations and in the presence of a constant 0.5 μM concentration of **asym6–6FAM**. Acquired values of *k*_1_ were plotted against [2-AET] to give a straight line passing through the origin (Figure S3B). Therefore, the 2-AET-**asym6–6FAM** reaction is overall second-order; first-order to **asym6–6FAM**, first-order to 2-AET. The second-order rate constant *k*_2_ of the reaction was derived from the slope of the line and is equal to 0.53 M^–1^ s^–1^. The *k*_2_ value is again similar to those acquired from the 2-AET reactions with two recently studied chemical probes.^7,15^ It is reasonable to assume that other thiols also follow the same second-order reaction mechanism when they react with **asym6–6FAM**.

We also performed pH titration studies on the 2-AET-**asym6–6FAM** reaction in order to determine the correlation between pH change and reaction rates in thiol- **asym6–6FAM** reactions. As we discussed in a recent study,^7^ FAM-related compounds are small-molecule dyes with fluorescence that is sensitive to pH changes and that is emitted in a neutral or basic environment when the deprotonated and quinoid FAM structures are being formed. Consequently, it was critical for the pH titration studies on the 2-AET-**asym6–6FAM** reaction to demonstrate that the increase of the 6-FAM fluorescence observed in the reaction was contributed primarily by the 2-AET-induced obliteration of contact fluorescence quenching in **asym6–6FAM** and not by the acid–base reactions of 6-FAM. We carefully chose buffers capable of maintaining a neutral or basic pH and subsequently obtained *k*_1_ for the reactions between the 6-FAM-containing **asym6–6FAM** (0.5 μM) and 2-AET (5 mM) in the presence of different buffers at pH values ranging from 6.5 to 9.0 (Figure S3C). The pH titration results unequivocally showed substantial increases of *k*_1_ values, while the pH of the buffers increased from 6.5 to 9.0. The values of *k*_1_ plateaued at approximately pH 8.5, which is characteristic of a general base catalysis mechanism and accounted for the acceleration of the 2-AET-**asym6–6FAM** reactions at alkaline pH. Nonlinear curve-fitting analysis of the pH titration results determined the p*K*_a1_ of 2-AET to be 8.20 ± 0.07, which is in excellent agreement with a reported p*K*_a1_ of 8.19.^19^

Finally, we carried out kinetic studies and demonstrated that the major free metal ions in biological fluids do not interfere in thiol-**asym6–6FAM** reactions and that the chemical probe **asym6–6FAM** is appropriate to use when determining the activity of biochemical reactions in biological fluids (Figure S3D). Similar to what we observed previously,^7^ both Cd^2+^ and Zn^2+^ inhibited the 2-AET-**asym6–6FAM** reaction. Moreover, Cu^2+^, Fe^3+^, Ni^2+^ and Co^2+^ formed complexes with **asym6–6FAM**, precipitated out of solution, and prevented our performing kinetic analysis of reactions in the presence of these cations. However, the metal ions that predominate in biological fluids such as blood – Na^+^, K^+^ and Mg^2+^ – did not affect the 2-AET-**asym6–6FAM** reactions. The results thus suggested that chemical probe **asym6–6FAM** could potentially be used to sensitively detect thiols in complex biological systems and that the **asym6–6FAM**-based analysis was rather inert to metal ions indigenous to biological fluids. In conclusion, the rapid, second-order thiol-**asym6–6FAM** reactions have the characteristics of a general base catalysis with an S_N_2 nucleophilic substitution reaction mechanism; moreover, metal ions commonly found in biological fluids did not inhibit the thiol-**asym6–6FAM** reactions.

### Harnessing **asym6–6FAM** to Measure Activity of BChE and PON1 Lactonase

The strong fluorogenic properties of **asym6–6FAM** encouraged us to exploit the chemical probe to determine the biomarker activity according to the reactivities of **asym6–6FAM** with various thiols. Here we sought to develop **asym6–6FAM**-based assays for sensitively measuring the activities of two important human biomarkers – BChE^16,20^ and PON1 ^15,21^ (Scheme 1). We measured BChE activity by following previously developed BChE activity assays under steady-state conditions^7,16,22^ except that the chemical probe **asym6–6FAM** was substituted for the less sensitive chemical probes used in previous studies (Scheme S4, SI). We anticipated that the fluorogenic properties of **asym6–6FAM** would allow us to study the time-dependent fluorescence turn-on of **asym6–6FAM** in the presence of standards of BChE from equine serum with different activities and to acquire the corresponding values of initial rate (*v*_i_) for **asym6–6FAM**-BChE-BTCh reactions in the steady state.^7,16^ We initially proceeded with the control reactions of tacrine inhibition on BChE catalysis in the presence of **asym6–6FAM** in order to confirm the hypothesis that the activity of BChE was essential to release the fluorescence of 6-FAM through thiol-mediated disulfide cleavage of **asym6–6FAM**. It is known that tacrine is a specific and competitive inhibitor of BChE activity in human plasma.^16,20,23^ Here tacrine inhibited the **asym6–6FAM**-BChE-BTCh reaction system and resulted in 6-FAM fluorescence levels being lower than those in the tacrine-free **asym6–6FAM**-BChE-BTCh reaction (Figure S6A, SI). In addition, either acetylcholinesterase or the serum protease thrombin was unable to cause a structural change in **asym6–6FAM** and to contribute to release of the 6-FAM fluorescence from **asym6–6FAM** (Figure S6B, SI). Emission of the 6-FAM fluorescence from **asym6–6FAM** depends indisputably on the activity of BChE catalysis. Linear regression analysis of BChE activity vs *v*_i_ from **asym6–6FAM**-BChE-BTCh reactions demonstrated that the **asym6–6FAM**-based assay provided a linear calibration with a slope *m* of 0.47 and a good linear detection range of 1.8–182.2 U L^–1^ (Figure 2A). Additionally, the **asym6–6FAM**-based assay was characterized with a limit of detection (LOD) of BChE activity equal to 1.06 U L^–1^, which was calculated by using the equation of LOD = 3*s*_b_/*m*.

**Scheme 1:**
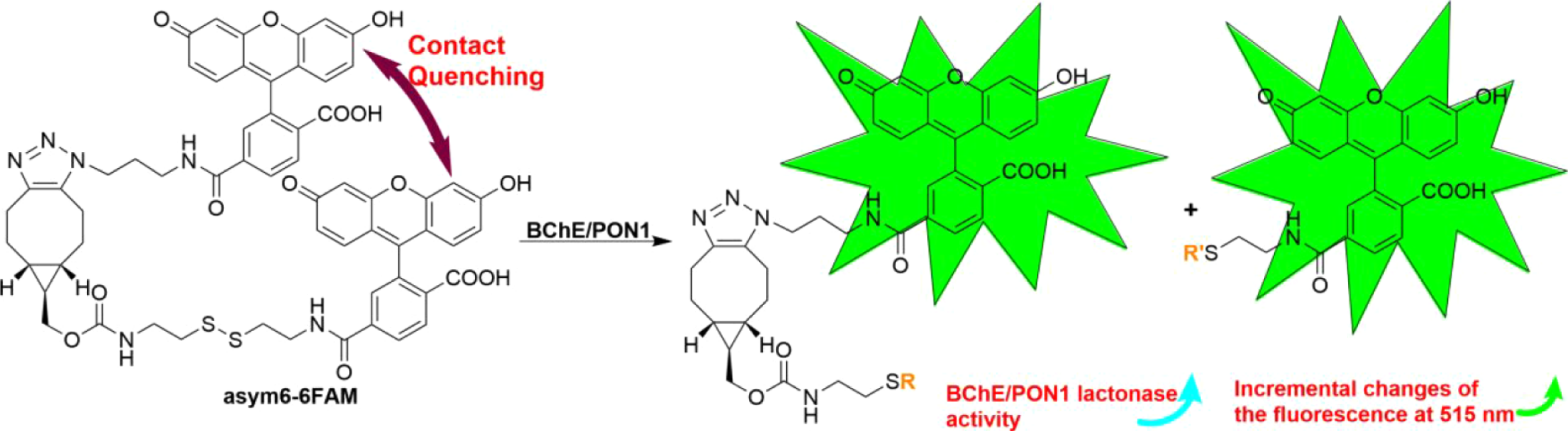
*Bis-*6-FAM-Containing Fluorescence Turn-on Chemical Probe **asym6–6FAM** for Sensitive Quantification of the Activities of BChE and PON1 Lactonase

**Figure 2:**
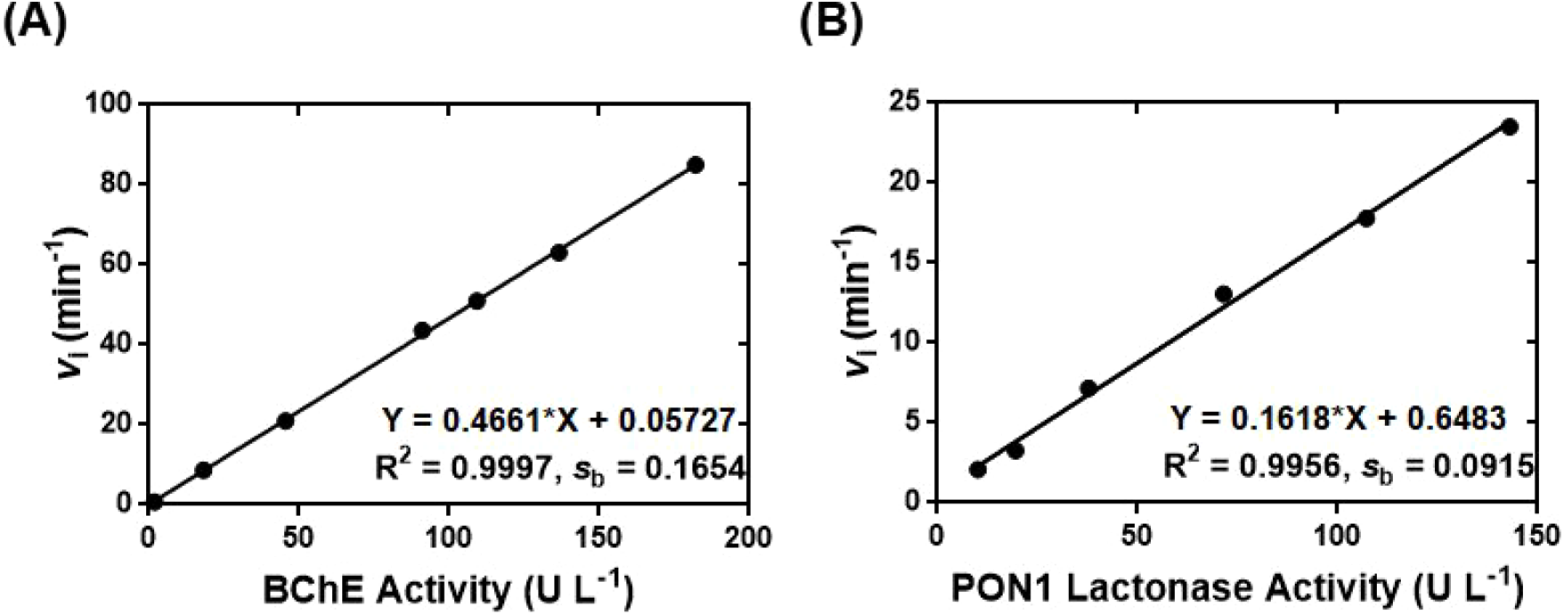
Fluorescence turn-on chemical probe **asym6–6FAM** for sensitively determining the enzyme activity of BChE and PON1 lactonase. (A) The **asym6–6FAM**-based fluorescence turn-on assay for quantifying BChE activity. (B) Utilization of **asym6–6FAM** to develop a fluorescence turn-on assay for determining PON1 lactonase activity.

Notably, the BChE activity assay built on the fluorogenic properties of **asym6–6FAM** presented a significant improvement over our previous BChE activity assays.^7,16^ Specifically, 25 μM of the EDANS-DABCYL-paired chemical probe were required in a BChE activity assay, while 0.5 μM of the *bis-exo*-BCN-based and *bis*-5-FAM-paired chemical probe were used in an assay for BChE activity.^7,16^ In contrast, the current assay required a much lower concentration (0.3 μM) of **asym6–6FAM** to sensitively determine the BChE activity in samples. Moreover, the **asym6–6FAM**-based BChE activity assay provided the lowest LOD value (LOD of 4.3 U L^–1^ for the EDANS-DABCYL-paired chemical probe and 1.9 U L^–1^ for the *bis-exo*-BCN-based and *bis*-5-FAM-paired chemical probe).^7,16^

Successfully utilizing **asym6–6FAM** to measure BChE activity (Figure 2A) allowed us to attain our goal of developing sensitive **asym6–6FAM**-based assays for measuring the PON1 lactonase activity. PON1 lactonase activity is the only catalytic reaction associated with the physiological functions of this important biomarker enzyme.^24^ We have written previously about the challenges and the urgency associated with developing assays capable of more sensitively quantifying PON1 lactonase activity in samples.^7^ Again, our laboratories were able to develop sensitive assays for measuring PON1 lactonase activity because we capitalized on our ability to purify lactonase-active, recombinant PON1 (rePON1) and to synthesize TBBL^15^ – the primary and specific substrate for determining PON1 lactonase activity.^25^

The current assay for PON1 lactonase activity (Schemes 1 and S5, SI) was also developed from our recently reported PON1 lactonase activity fluorescence assays under steady-state conditions and was conducted by substituting **asym6–6FAM** for the chemical probes used in previous PON1 lactonase activity measurements.^7,15^ Similarly, we began with the control reactions of the **asym6–6FAM**-PON1-TBBL system with 2-hydroxyquinoline (2-HQ) in order to confirm the requirement of PON1 catalysis in detecting the 6-FAM fluorescence released by nucleophilic attacks of thiols on **asym6–6FAM**. As a specific and competitive inhibitor of PON1 catalysis,^15,24^ 2-HQ was proposed to also inhibit PON1 catalysis in the presence of **asym6–6FAM**.

Consistent with previous reports, 2-HQ competitively inhibited the **asym6–6FAM**-PON1-TBBL system, and 6-FAM fluorescence levels were lower than those of the 2-HQ-free **asym6–6FAM**-PON1-TBBL reaction (Figure S6C, SI). Therefore, we concluded that the unveiling of fluorescence emission of **asym6–6FAM** required the prior action of the PON1 enzyme toward the TBBL substrate. The chemical probe **asym6–6FAM** was further employed to develop a sensitive assay for determining the lactonase activity of PON1 (Figure 2B). Kinetic studies of the time-dependent fluorescence turn-on of **asym6–6FAM** in the presence of rePON1 standards^26^ with different activities in the steady state facilitated the determination of the corresponding values of *v*_i_. The linear regression analysis of PON1 lactonase activity vs *v*_i_ revealed that the fluorescence turn-on assay built on **asym6–6FAM** provided a linear calibration with a detection range of 10.1–143.0 U L^–1^ and an LOD of 1.7 U L^–1^ (Figure 2B). The **asym6–6FAM**-based assay for determining lactonase activity of PON1 thus provided a lower LOD of 1.7 U L^–1^ but a narrower dynamic detection range than our previously reported fluorescence assays for PON1 lactonase activity.^7,15^ Therefore, the novel fluorescence assay based on the fluorogenic properties of **asym6–6FAM** is a sensitive method for measuring the lactonase activity of PON1.

Figures 1 and 2 offer evidence for the ability of the chemical probe **asym6–6FAM** to release two equiv of the 6-FAM fluorescence from the chemical probe and to provide sensitive assays for determining activity of BChE and PON1 lactonase. This suggested that the **asym6–6FAM**-based fluorescence assays had the potential to accurately measure the activity of BChE and PON1 lactonase in serum samples and to be used to develop methods for screening BChE inhibitors. The following three sections detail the results of our attempts to analyze clinical samples using the **asym6–6FAM**-based fluorescence assays in the manner indicated above.

### The **asym6–6FAM**-Based Fluorescence Turn-On Assays for Sensitively and Accurately Determining Activity of BChE and PON1 Lactonase in Human Serum

We attempted to corroborate the usefulness of the **asym6–6FAM**-dependent fluorescence turn-on assays for sensitively and accurately determining the activities of BChE and PON1 lactonase in human serum (Figure 3). We first performed control experiments and demonstrated that interactions of serum proteins with **asym6–6FAM** and activities of serum biothiols including GSH and of serum enzymes other than BChE and PON1 did not affect the accurate determination of BChE and PON1 lactonase activity in the **asym6–6FAM**-based assays (Figure S7, SI). This conclusion was supported by the facts that BChE and PON1 are the major esterases in human plasma.^18^ In addition, it is well documented that BTCh is a specific substrate used for determining BChE activity in blood samples^27,28^ and that tacrine is a specific and competitive inhibitor of BChE activity in human plasma.^16,20,23^ Moreover, past studies have confirmed that TBBL is an appropriate substrate for specifically determining PON1 activity in serum^25^ and that 2-HQ is a specific, competitive inhibitor of PON1 catalysis.^24^

**Figure 3:**
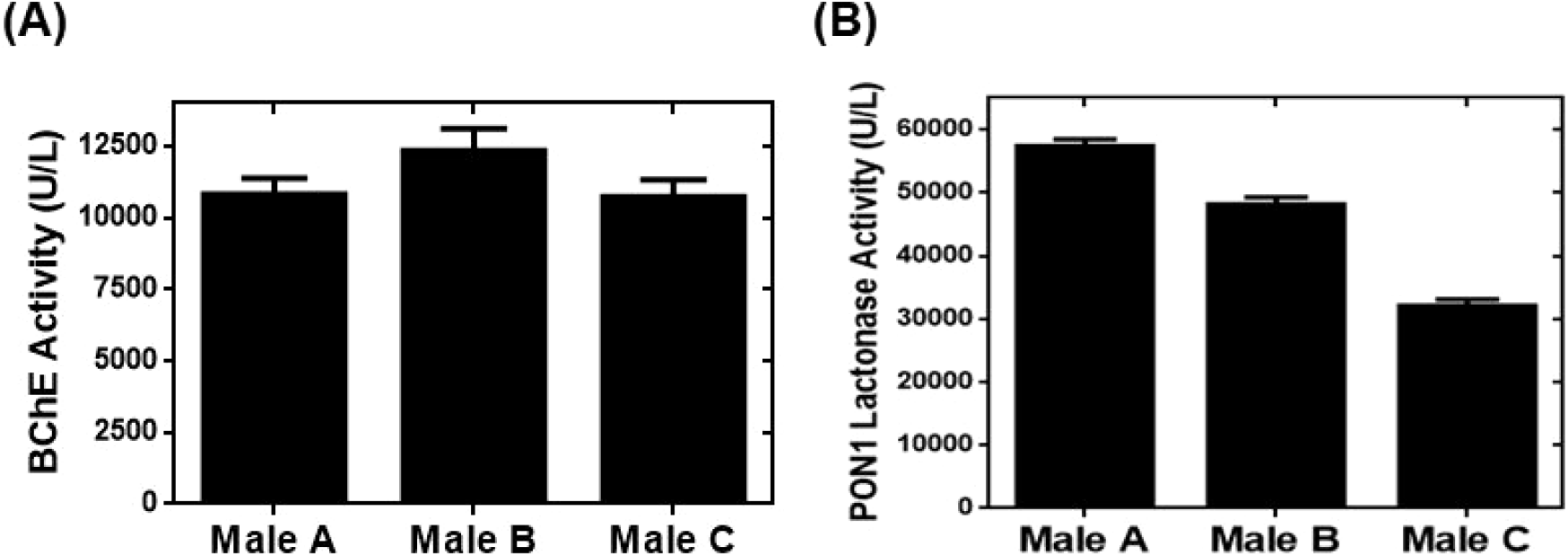
**asym6–6FAM**-based assays for determining the activity of BChE and PON1 lactonase in human serum. (A) BChE activity in serum samples from three healthy males. (B) PON1 lactonase activity in serum samples from three healthy males.

We later exploited the linear regression equation reported in Figure 2A to determine BChE activity in the serum from three healthy males. The **asym6–6FAM**-based assay successfully quantified BChE activity in the serum samples (Figure 3A) and provided an average BChE activity of 11,155 ± 902 U L^–1^. Both the average and individual values of BChE activity were within the normal physiological range of BChE activity of 5,900–13,200 U L^–1^.^29^ BChE activity in the serum samples was also determined by the standard Ellman’s assay,^30^ which provided results similar and comparable to those reported in Figure 3A (Figure S8A, SI). Biothiols such as GSH and cysteine to interfere with the **asym6–6FAM**-based assay were unlikely to be an issue because the assay protocol included a step to eliminate biothiol interference, and the protocol was followed exactly as written in Experimental (SI). Consequently, most of the endogenous biothiols in human serum that might have interfered with the assay was consumed before the 6-FAM fluorescence was measured kinetically in the current assays. The data demonstrated that the fluorescence turn-on assay based on **asym6–6FAM** can directly and accurately determine BChE activity in serum samples that contain physiological levels of GSH and other serum biothiols.

To determine PON1 lactonase activity in serum, we employed the equation acquired by performing a linear regression using the calibration curve in Figure 2B and quantified PON1 lactonase activity in serum samples from the same three healthy males. The **asym6–6FAM**-based assay quantified PON1 lactonase activity in the serum samples (Figure 3B) to be in the range of 31,500–56,800 U L^–1^ with an average value of 45,300 ± 12,800 U L^–1^. In comparison, PON1 lactonase activity in human serum was previously reported to be 3,800 ± 1,900 U L^–1^ according to a colorimetric Ellman’s assay^31^ and 17,800–19,500 U L^–1^ determined by our recently reported fluorescence turn-on assay.^15^ The **asym6–6FAM**-based assay for quantifying PON1 lactonase activity in human serum provided values that were over an order of magnitude higher than those obtained from the colorimetric Ellman’s assay and more than double those determined by our previously reported fluorescence turn-on assay. The discrepancy between the values of PON1 lactonase activity in human serum could be attributed to the fact that Ellman’s assay and our past fluorescence assay are less sensitive than the fluorescence turn-on assay based on **asym6–6FAM**. Consequently, we did not perform Ellman’s assay for the serum samples from the three healthy males because of the expectation of significantly different values of PON1 lactonase activity obtained from Ellman’s assay and the **asym6–6FAM**-based assay. Overall, this suggests that the **asym6–6FAM**-based fluorescence assay may more sensitively and accurately determine the PON1 lactonase activity in serum samples.

### The Fluorescence Turn-On Assay Built on **asym6–6FAM** Was Effective for Kinetic Analysis of BChE Inhibition

We further explored the potential of the assay based on **asym6–6FAM** to be used to measure the inhibition of BChE catalysis and to develop methods for screening BChE inhibitors useful in drug discovery.^16^ Here we employed the **asym6–6FAM**-based assay to study the inhibition of tacrine in BChE catalysis. As shown in Figures S6A and S7A, tacrine clearly inhibited the BChE-BTCh reaction competitively in the presence of **asym6–6FAM** and resulted in 6-FAM fluorescence levels being lower than in the tacrine-free **asym6–6FAM**-BChE-BTCh reaction. We showed that *v*_i_ gradually decreased as tacrine concentrations increased in the assay based on **asym6–6FAM** (Figures 4A and S9, SI). Kinetic analysis of tacrine inhibition on BChE catalysis facilitated our graphing a Dixon plot (Figure 4B) and determining the *K*_i_ value of 6.29 nM, which was relatively close to the *K*_i_ value of 8.70 ± 1.17 nM determined by Ellman’s method.^23^ These results supported the idea that the **asym6–6FAM**-based fluorescence assay might be an appropriate platform for discovering novel BChE inhibitors that could prove useful in diagnosing and treating BChE-related human diseases.^16^

**Figure 4:**
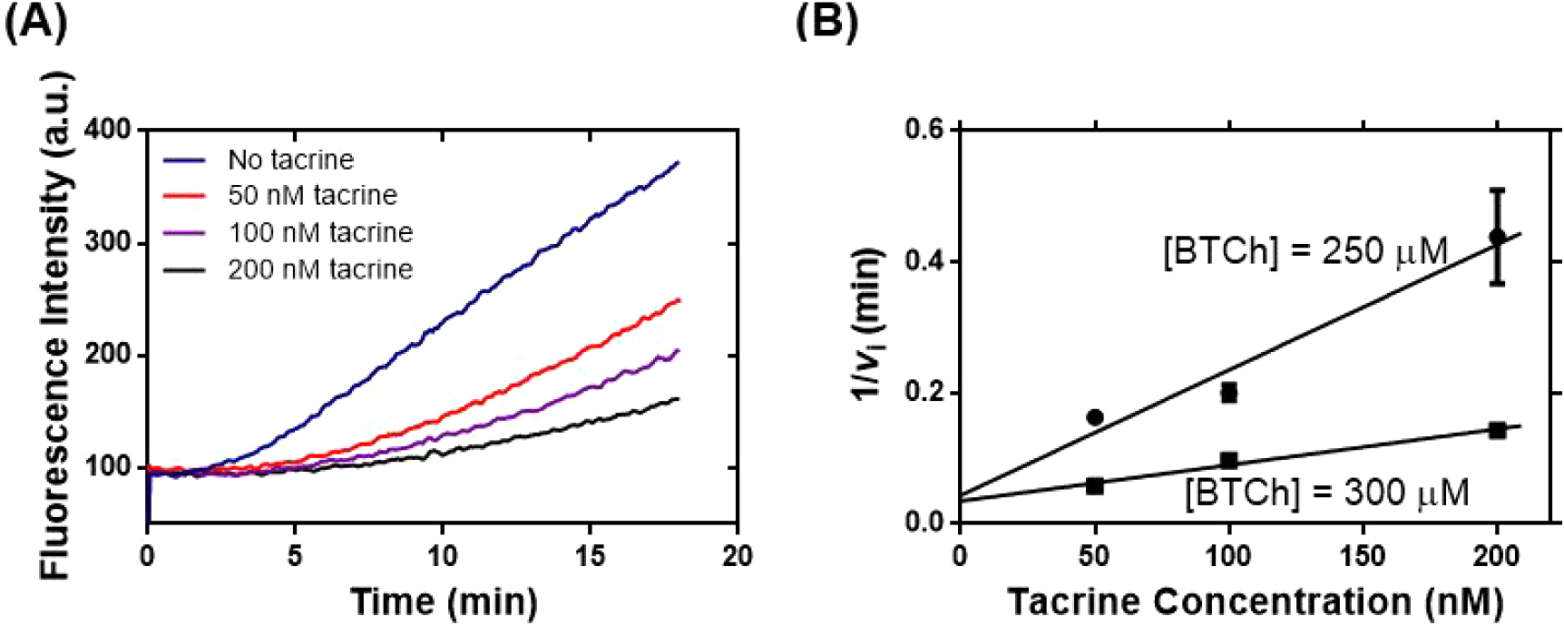
Tacrine inhibition of BChE catalysis was determined by the fluorescence assay based on **asym6–6FAM**. (A) Representative time-course kinetic studies of tacrine inhibition of BChE (136.7 U L^–1^) catalysis were performed in the presence of **asym6–6FAM** (0.3 μM), BTCh (250 μM), and tacrine (0, 50, 100, or 200 nM) in PB. (B) Dixon plot of the inhibitory kinetics of tacrine on BChE catalysis constructed from data found in Figures 4A and S9.

### High-Throughput Assay Based on **asym6–6FAM** for Accurately Determining BChE Activity in Human Serum

Several fluorescence probes have recently been reported as novel ways to measure BChE activity.^7,16^ None of the probes, however, has been deployed to develop high-throughput assays for quantifying BChE activity in biological samples. A low sensitivity, colorimetric 96-well assay based on wax-printed Prussian Blue paper was reported to measure BChE activity in the 2,000–15,000 U L^–1^ range with a LOD of 800 U L^–1^ and was exploited to determine BChE activity in human serum.^29^ There is thus an urgent demand for more sensitive and accurate high-throughput assays based on fluorescence measurements for quantifying BChE activity in biological fluids. Successfully developing the **asym6–6FAM**-dependent and fluorescence spectrometer-based assay for determining BChE activity (Figures 2A and 3A) emboldened us to modify the assay for high-throughput formats capable of quantifying BChE activity in biological fluids such as human serum.

We successfully developed a high-throughput assay built on **asym6–6FAM** for determining BChE activity under steady-state conditions (Figures 5A and S10, SI). We again performed linear regression analysis of BChE activity vs *v*_i_ from **asym6–6FAM**-BChE-BTCh reactions and demonstrated that the high-throughput **asym6–6FAM**-based assay provided a linear calibration with a slope *m* of 1.82 and a linear detection range of 10.0–182.2 U L^–1^. The high-throughput **asym6–6FAM**-based assay was further characterized by calculating the LOD of BChE activity to be 0.95 U L^–1^ using the equation LOD = 3*s*_b_/*m*. In addition, the calibration equation from linear regression in Figure 5A facilitated our determination of BChE activity in the serum samples from the same three healthy males described in Figure 3. The high-throughput **asym6–6FAM**-based assay successfully quantified BChE activity in the serum samples (Figure 5B) and provided an average BChE activity of 11,027 ± 719 U L^–1^. As was the case previously, both the average and individual values of BChE activity were within the normal physiological range of BChE activity (5,900–13,200 U L^–1^)^29^ and were consistent with those obtained from the fluorescence spectrometer-based assay (Figure 3A). Additionally, BChE activity in the serum samples was also determined by a high-throughput assay modified from the standard Ellman’s analysis^30^ and was confirmed to provide results similar and comparable to those described in Figure 5B (Figure S8B, SI). The high-throughput **asym6–6FAM**-based assay was also less susceptible to interference from serum biothiols such as GSH because endogenous biothiols in human serum was consumed before kinetic measurements of the 6-FAM fluorescence using the high-throughput assay built on **asym6–6FAM** were performed. The high-throughput **asym6–6FAM**-based fluorescence turn-on assay is thus useful for directly and accurately determining the BChE activity in a large number of serum samples.

**Figure 5:**
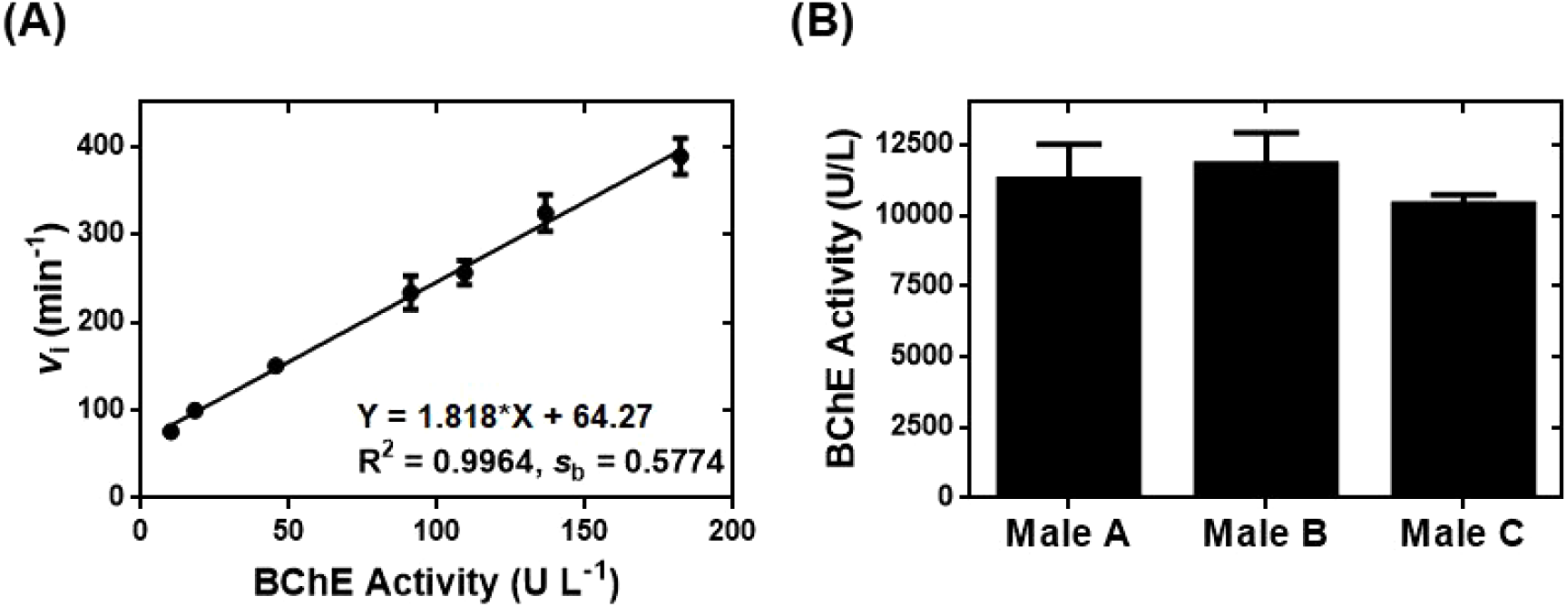
High-throughput **asym6–6FAM**-based assay for sensitively determining the activity of BChE in human serum. (A) Development of a high-throughput fluorescence turn-on assay built on **asym6–6FAM** to quantify BChE activity. (B) BChE activity determined by high-throughput analysis of the same human serum samples used for the experiment reported in Figure 3.

## Conclusions

This study has demonstrated a critical use for contact quenching in a sensitive, dual-labeled, profluorescent chemical probe synthesized from a *mono exo*-BCN-derivatized cystamine framework and designated as **asym6–6FAM**. The **asym6–6FAM**-based, fluorescence spectrometer-based fluorescence turn-on assays were successfully developed and exploited to determine the activities of BChE and PON1 lactonase in three human serum samples (Figure 3). The **asym6–6FAM**-dependent assays were adapted for a high-throughput format, which efficiently quantified BChE activity in a large number of biological samples (Figures 5 and S10). The high-throughput, profluorescent assay built on **asym6–6FAM** is, to the best of our knowledge, the first contact quenching-based method for quantifying BChE activity in a large number of biological samples. Since aberrant activity of BChE has been closely associated with the development and progression of a range of human diseases,^16^ successfully using the high-throughput **asym6–6FAM**-based fluorescence turn-on assay to determine BChE activity in multiple human serum samples (Figure 5) is expected to have broad applications in clinical and basic research. We are also working to develop high-throughput **asym6–6FAM**-based fluorescence assays for quantifying PON1 lactonase activity in biological fluids. Moreover, we are synthesizing more thiol-incorporated substrate molecules for different substrate specificity applications in order to expand the **asym6–6FAM**-dependent assays able to determine the hydrolytic activity of other clinically important enzymes.

This research affirmed an essential connection between contact quenching and the spectroscopic properties of the synthesized, dual-labeled, profluorescent chemical probes derived from a *mono exo*-BCN-containing cystamine framework and provided vital information about the structural requirements of effective contact quenching in profluorescent constructs. Our contributions concerning contact quenching in profluorescent chemical probes will help pave the way for designing assays capable of performing diverse chemical analyses with greater efficiency and for synthesizing novel profluorescent constructs and materials with even broader applications.

## Supplementary Material



## Notes

Materials and methods, references in the text, glossary, and NMR and HRMS spectra for compounds (**asym5–5FAM**, **asym5–6FAM**, **asym6–5FAM**, and **asym6–6FAM**) (PDF)
