# Supplementary material for: Harnessing Contact-Quenched, Profluorescent Chemical Probes for Sensitive Determination and High-Throughput Measurements of Enzyme Activity
Source: ACS Omega. 2025 Nov 22;10(48):58566–76. doi: 10.1021/acsomega.5c06578 (PMC12771411; doi:10.1021/acsomega.5c06578)
Supplement: Supplementary file 1 [file ao5c06578_si_001.pdf]

Supporting Information

Harnessing contact-quenched, profluorescent chemical probes for sensitive determination and high-throughput measurements of enzyme activity

Chien-Hui Huang,[a,§] Chia-Yen Dai[b,c,§] Su-Hung Wang,[d,§] Scott Severance,[e]

Chi-Ching Hwang,[f] Yu-Chen Liu,[a] Bao-Lin Yeh,[a] Yung-Chieh Weng,[a] Siao-Lei Yu,[a]

Hsing-Tao Kuo,[d] Li-Fang Wang,[a] Jeh-Jeng Wang,[a] and Tzu-Pin Wang,[a,g,*]

* Corresponding author: tzupinw@kmu.edu.tw

* Contact address: Department of Medicinal and Applied Chemistry and Kaohsiung Medical University Hospital, Kaohsiung Medical University, Kaohsiung, 80708, Taiwan.

Contents

1. Schemes S1-S5 ……..…………………………..…………………....…S3-S7

2. Figures S1-S22 .... S8-S32

3. Table S1 ……………………………………………………..………..S33

4. Experimental ……….. S34-S43

5. References S44

6. Glossary ……………………………………………...…………………….S45

Schemes

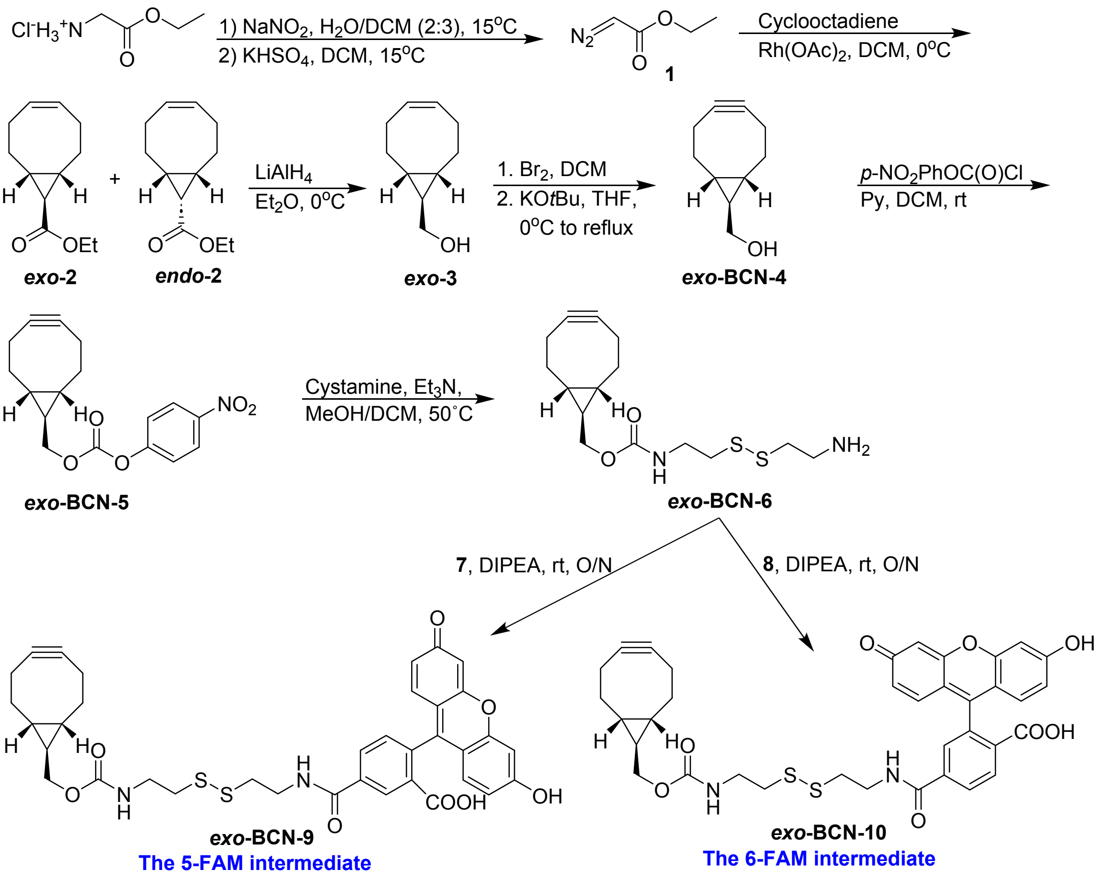

Scheme S1. Synthesis of the bicyclononyne (BCN) ***exo*-BCN-4** and derivatives of ***exo*-BCN-4**. The method of Rady *et al.* was adopted and modified in order to synthesize ethyl diazoacetate (**1**).[1] Syntheses of ***exo-*/*endo*-2** and ***exo*-3** to ***exo*-BCN-10** were reported previously.[2-5]

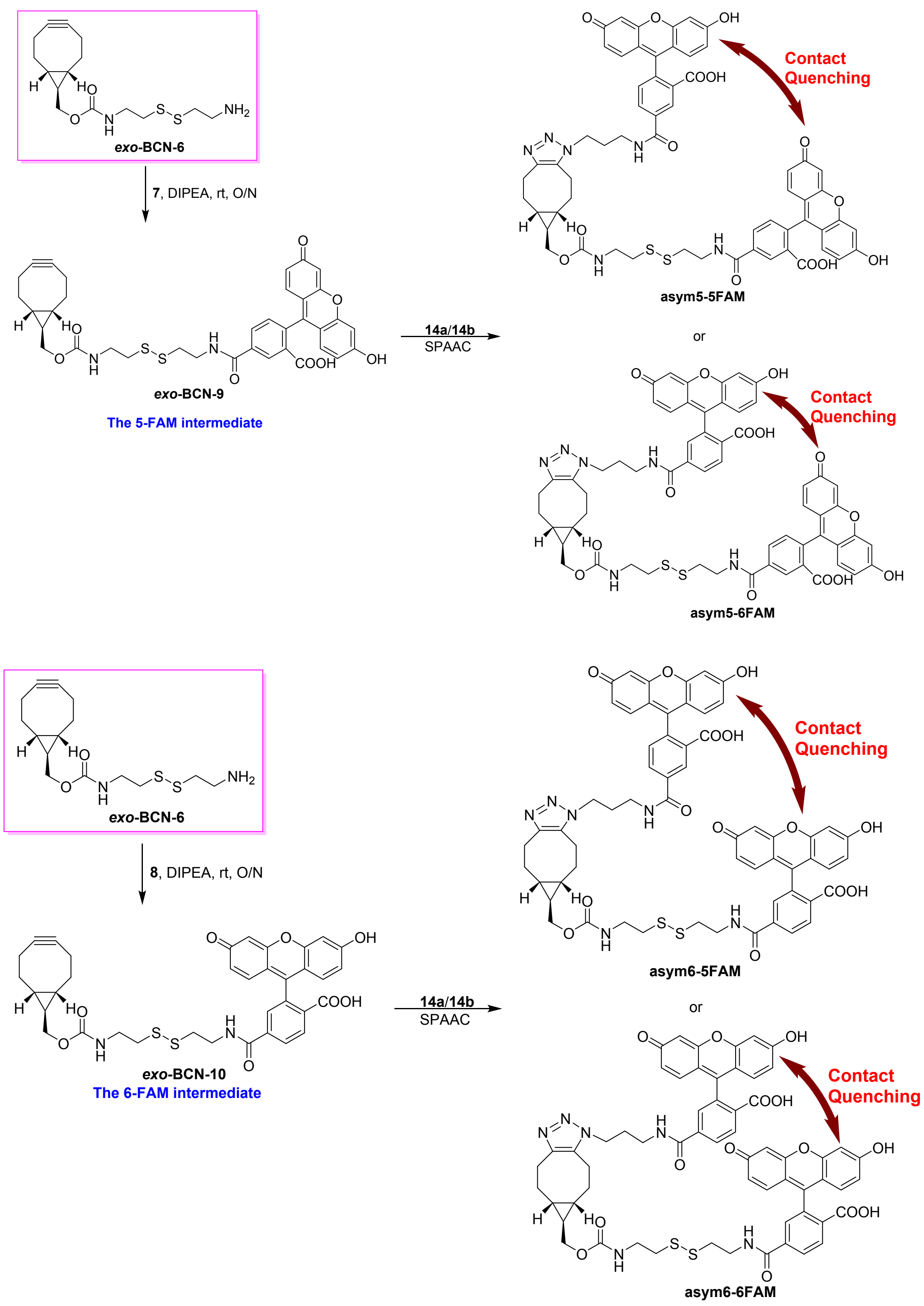

Scheme S2. Harnessing the critical steric properties of ***exo*-BCN-6**, highlighted by the pink box, to synthesize the non-symmetrical and *bis*-carboxyfluorescein (FAM)-containing fluorescence turn-on chemical probes **asym5-5FAM**, **asym5-6FAM**, **asym6-5FAM** and **asym6-6FAM**. In the SPAAC reactions,

***exo*-BCN-9** or ***exo*-BCN-10** was reacted with **14a**/**14b** in 1,4-dioxane under reflux for 6 h. We reported the syntheses of ***exo*-BCN-6**,**7**, **8** and **14a**/**14b** previously [4, 5].

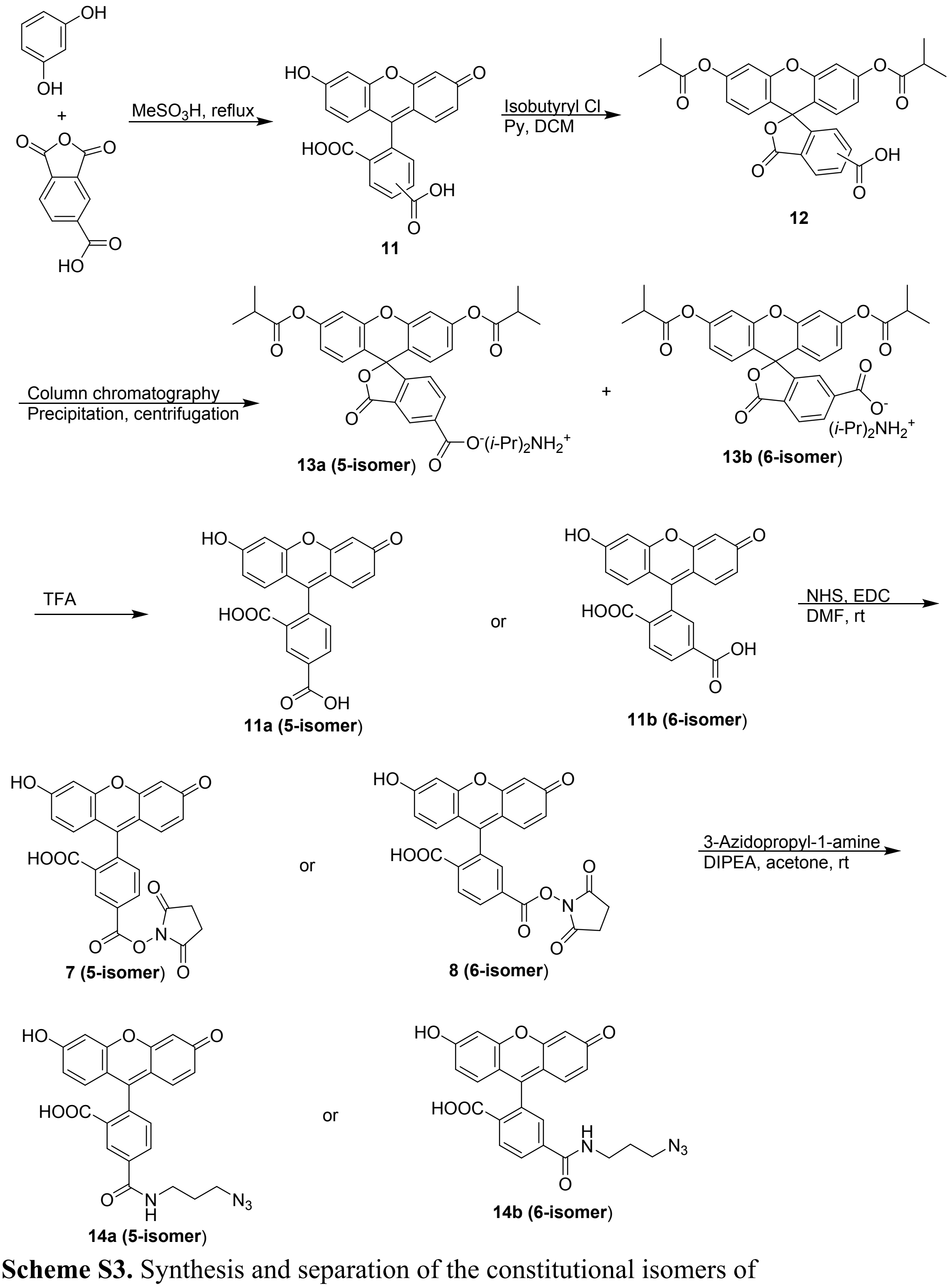

Scheme S3. Synthesis and separation of the constitutional isomers of carboxyfluorescein (FAM) (**11**) and the corresponding derivatives **7-8** and **12-14**.[4, 5] Synthesis of 5(6)-carboxyfluorescein (**11**) was achieved by generally following the method of Lee and Grissom.[6]

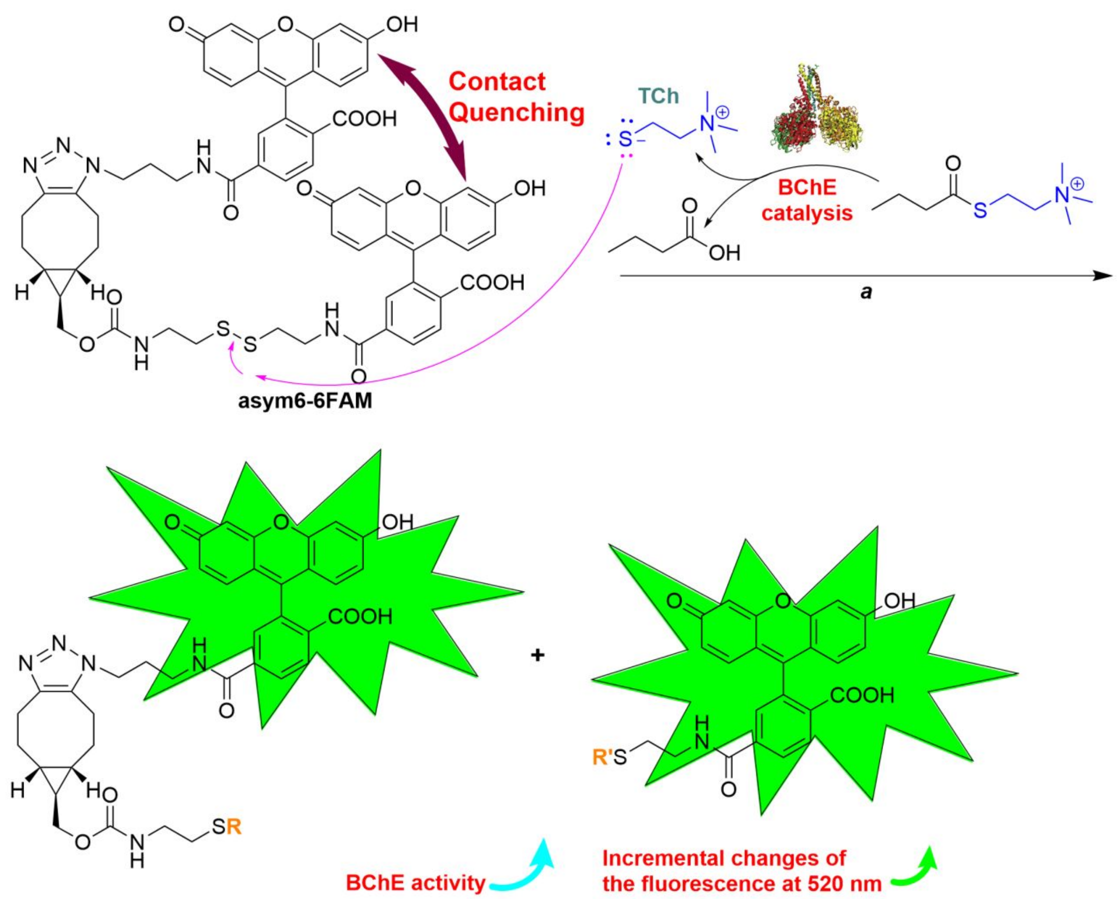

Scheme S4. An application of the chemical probe **asym6-6FAM** to quantitatively determine activity of BChE. ***a***: phosphate buffer (PB; 100 mM, pH 7.4), 37˚C. Thiocholine (TCh) was one of the products of BChE catalysis, and its structure is highlighted in blue. The thiolate of TCh subsequently participated in nucleophilic attack on the disulfide bond in **asym6-6FAM** to obliterate the contact quenching effect and to release the 6-carboxyfluorescein (6-FAM) fluorescence. An increase in 6-FAM fluorescence levels (green arrow) is a direct result of and proportional to increased BChE activity (cyan arrow). R and R’: a hydrogen or the thiolate of TCh.

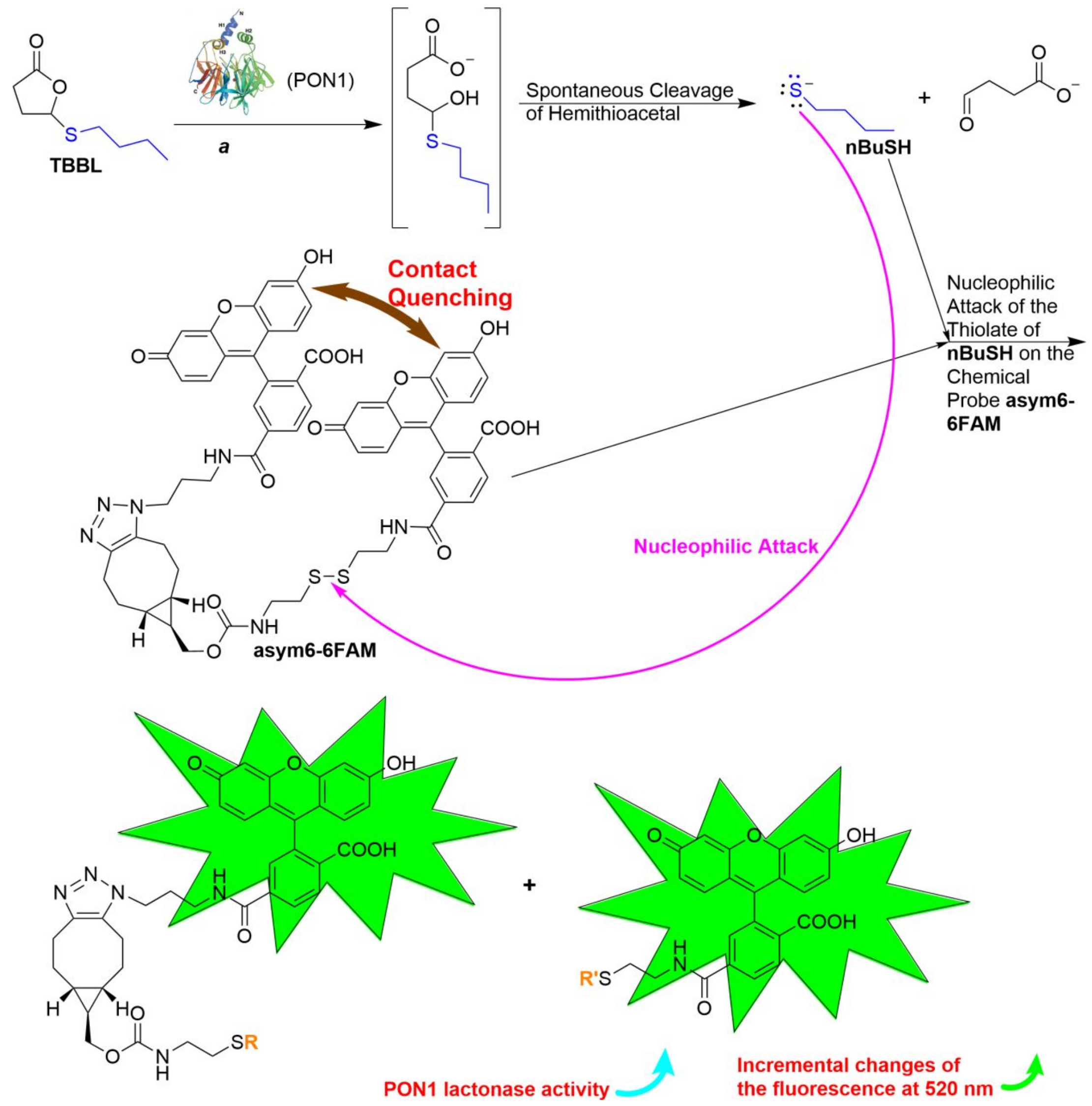

Scheme S5. Use of the chemical probe **asym6-6FAM** to quantitatively determine paraoxonase 1 (PON1) lactonase activity. PON1-catalyzed hydrolysis of 5-thiobutyl butyrolactone (TBBL, a sulfur-containing lactone) provides a labile hemithioacetal, which spontaneously cleaves to yield 1-butanethiol (nBuSH) as one of the products. The thiolate of nBuSH is highlighted in blue. Subsequently, the thiolate carried out a nucleophilic attack (pink arrow) on the disulfide bond in **asym6-6FAM** to obliterate the fluorescence quenching effects (double arrowed brown curve) and to release the 6-FAM fluorescence. Increased levels of the 6-FAM fluorescence (green arrow) are, therefore, a direct result of and proportional to increases in PON1 lactonase activity (cyan arrow). ***a***: (TBBL), Tris, Ca^{2+}, pH 8.0. R and R’: a hydrogen or the thiolate of nBuSH.

Figures.

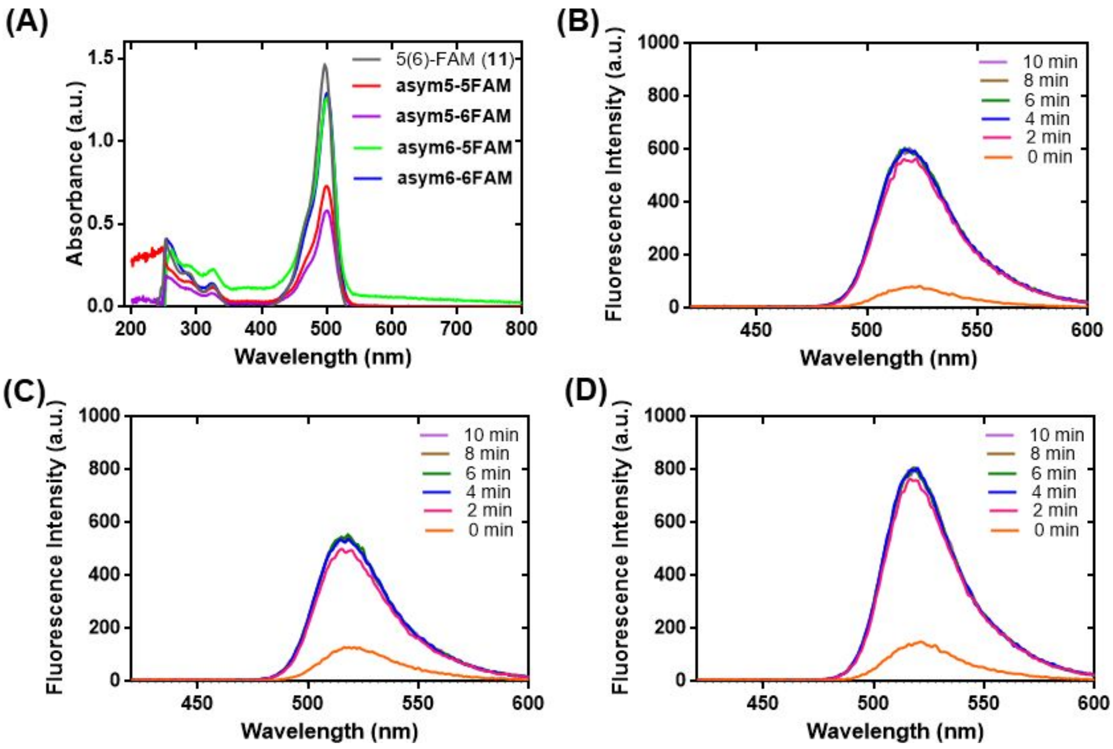

Figure S1. Contact quenching of the four non-symmetrical *bis*-FAM chemical probes and release of intrinsic fluorescence in three of the four chemical probes as determined by studies of UV-Vis spectrophotometry and fluorescence measurements, respectively. (A) UV-Vis spectra of the four non-symmetrical *bis*-FAM chemical probes (10 μM each) and **11** (20 μM). (B) The time-dependent increase of the 5-carboxyfluorescein (5-FAM) fluorescence in a reaction of **asym5-5FAM** (0.5 μM) with 2-aminoethanethiol (2-AET) (50 mM) in phosphate buffer (PB; 100 mM, pH 7.4). (C) The time-dependent increase of FAM fluorescence in a reaction of **asym5-6FAM** (0.5 μM) with 2-AET (50 mM) in PB. (D) The time-dependent increase of FAM fluorescence in a reaction of **asym6-5FAM** (0.5 μM) with 2-AET (50 mM) in PB.

(A)

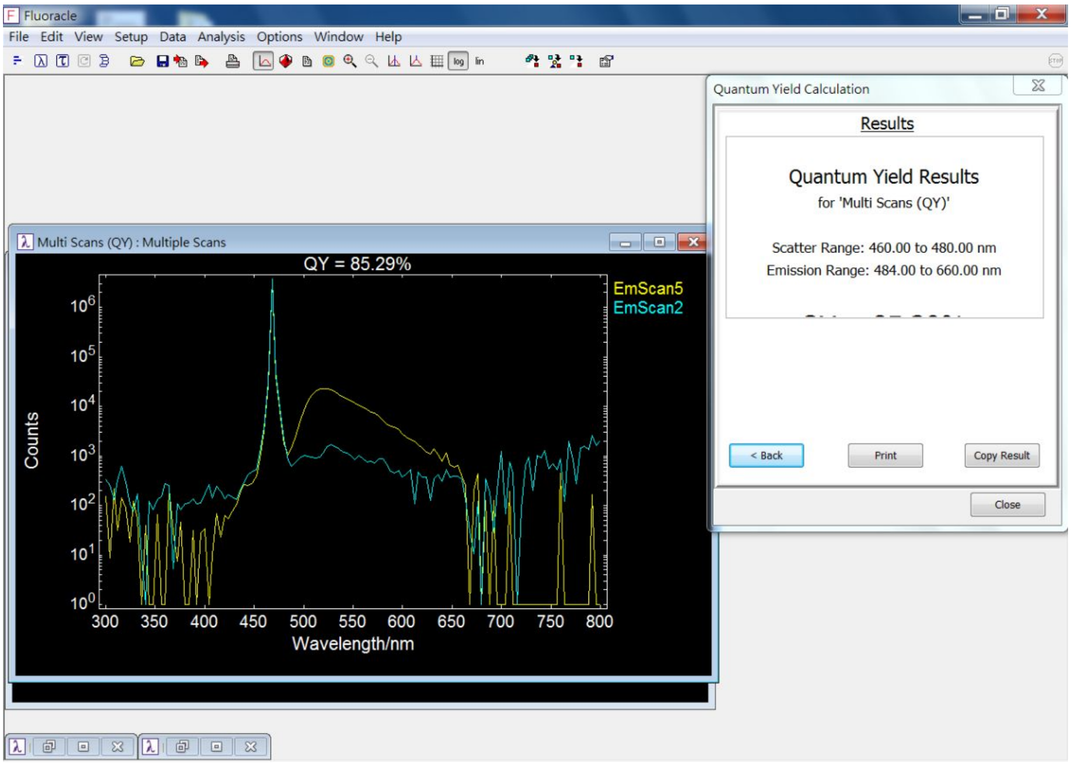
Fluoracle
File Edit View Setup Data Analysis Options Window Help
Multi Scans (QY) : Multiple Scans
QY = 85.29%
EmScan5
EmScan2
Counts
Wavelength/nm
Quantum Yield Calculation
Results
Quantum Yield Results
for 'Multi Scans (QY)'
Scatter Range: 460.00 to 480.00 nm
Emission Range: 484.00 to 660.00 nm
< Back
Print
Copy Result
Close

(B)

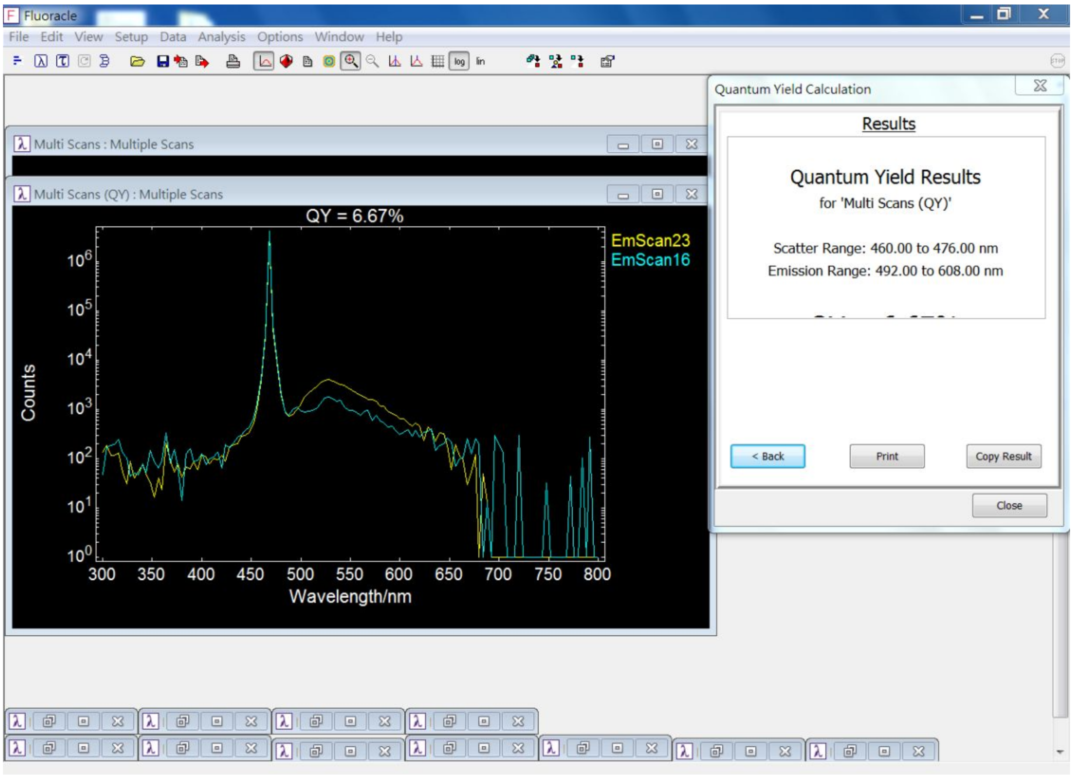
Fluoracle
File Edit View Setup Data Analysis Options Window Help
Multi Scans : Multiple Scans
Multi Scans (QY) : Multiple Scans
QY = 6.67%
EmScan23
EmScan16
Counts
Wavelength/nm
Quantum Yield Calculation
Results
Quantum Yield Results
for 'Multi Scans (QY)'
Scatter Range: 460.00 to 476.00 nm
Emission Range: 492.00 to 608.00 nm
< Back
Print
Copy Result
Close

(C)

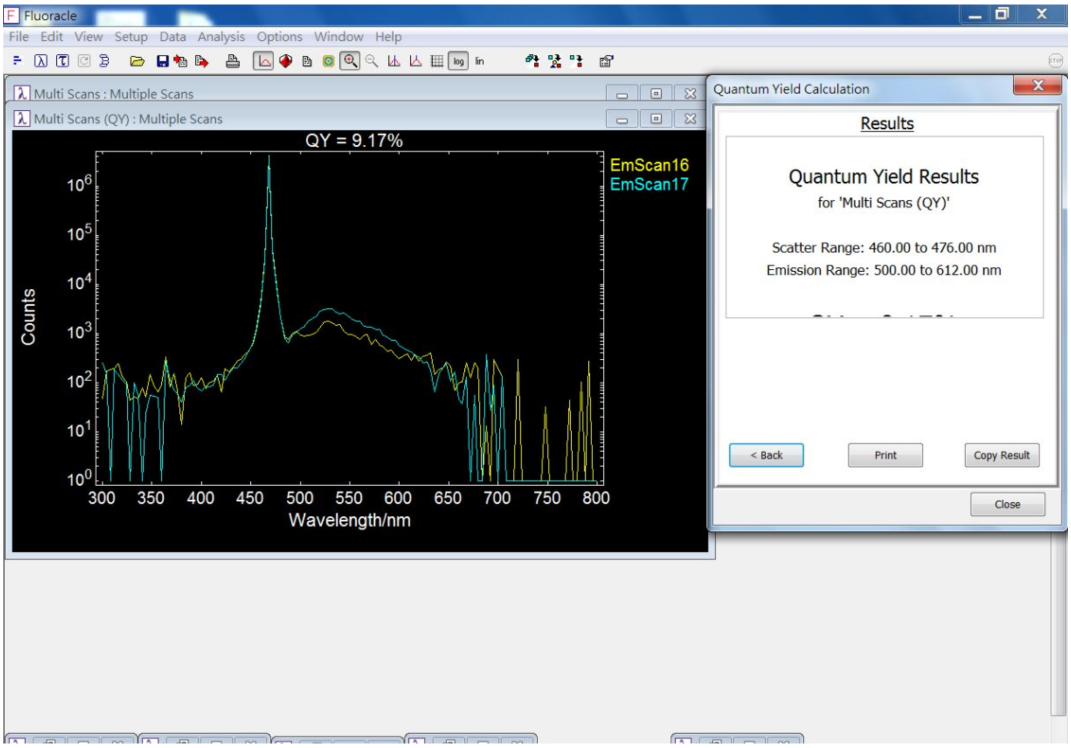
Fluoracle
File Edit View Setup Data Analysis Options Window Help
Multi Scans : Multiple Scans
Multi Scans (QY) : Multiple Scans
QY = 9.17%
EmScan16
EmScan17
Counts
Wavelength/nm
Quantum Yield Calculation
Results
Quantum Yield Results
for 'Multi Scans (QY)'
Scatter Range: 460.00 to 476.00 nm
Emission Range: 500.00 to 612.00 nm
< Back
Print
Copy Result
Close

(D)

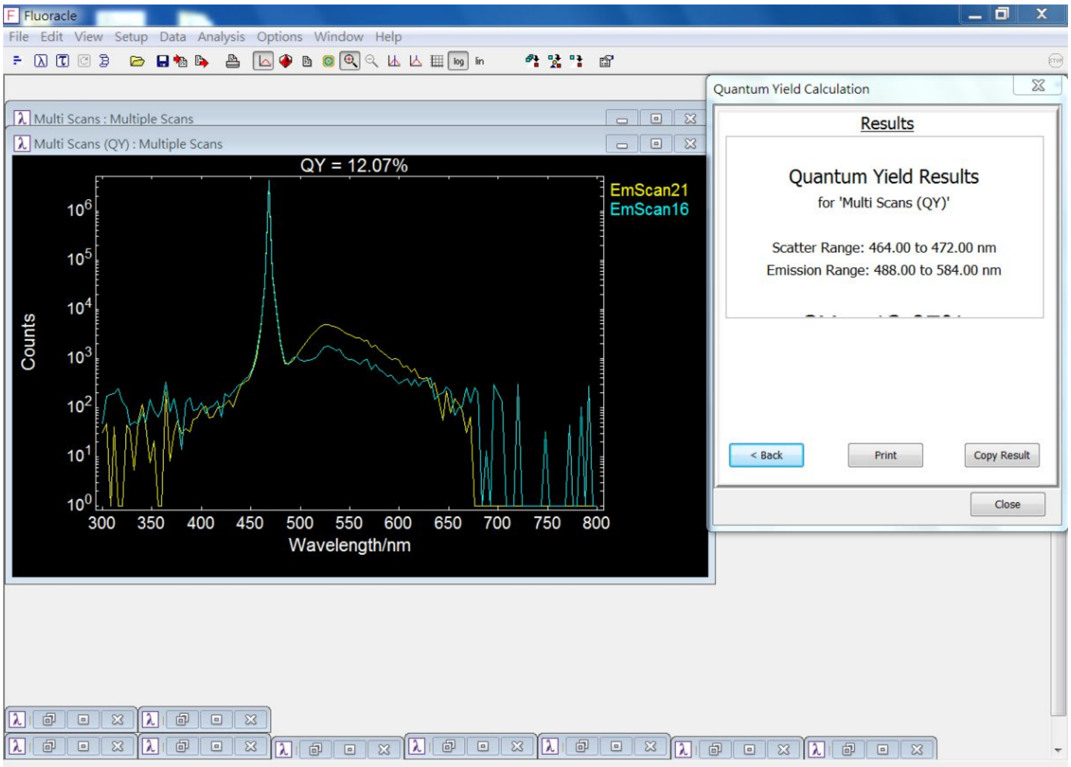
Fluoracle
File Edit View Setup Data Analysis Options Window Help
Multi Scans : Multiple Scans
Multi Scans (QY) : Multiple Scans
QY = 12.07%
EmScan21
EmScan16
Counts
Wavelength/nm
Quantum Yield Calculation
Results
Quantum Yield Results
for 'Multi Scans (QY)'
Scatter Range: 464.00 to 472.00 nm
Emission Range: 488.00 to 584.00 nm
< Back
Print
Copy Result
Close

(E)

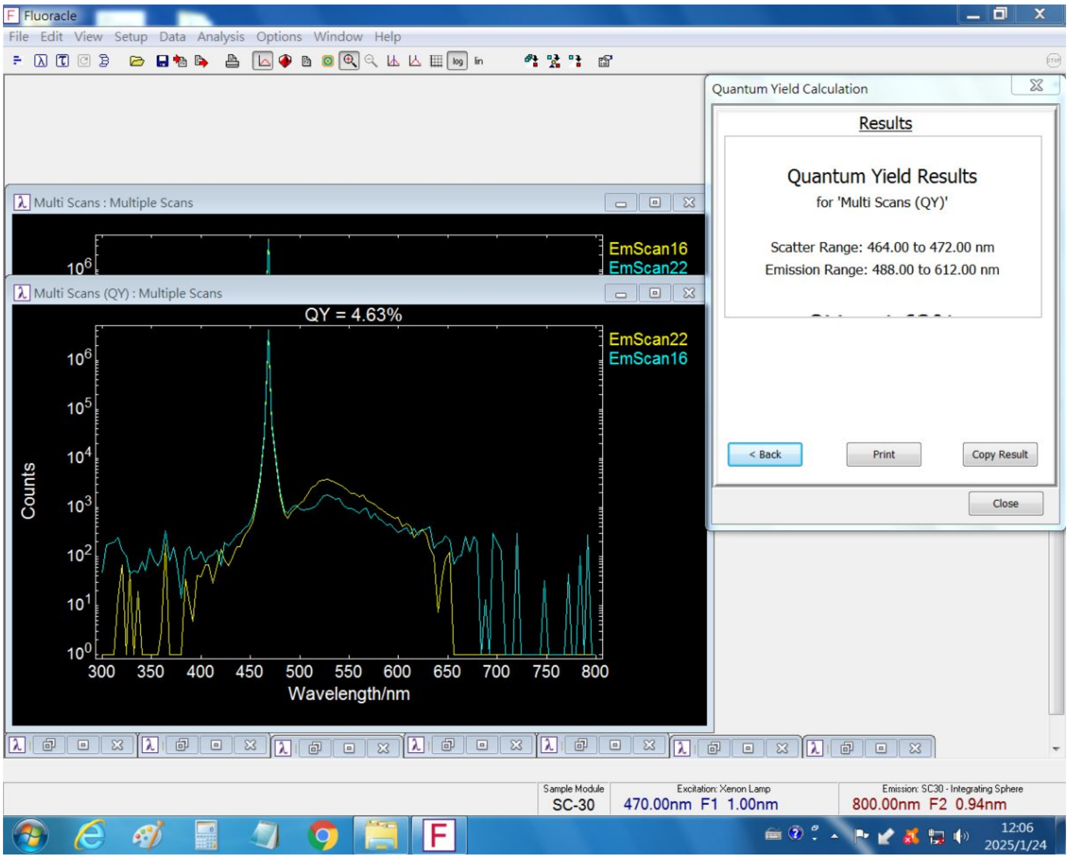

Figure S2. Determination of absolute quantum yield (Φ_A) for (A) **11** (1 μM) in phosphate buffer (PB; 100 mM, pH 7.4), (B) **asym5-5FAM** (0.5 μM) in PB, (C) **asym5-6FAM** (0.5 μM) in PB, (D) **asym6-5FAM** (0.5 μM) in PB, and (E) **asym6-6FAM** (0.5 μM) in PB. Measurements of Φ_A were achieved by using a FS5 Spectrofluorometer (Edinburgh Instruments, UK) equipped with a SC-30 150 mm diameter integrating sphere. Values of Φ_A were calculated by following the instructions of the manufacturer. The excitation wavelength was 470 nm in this study.

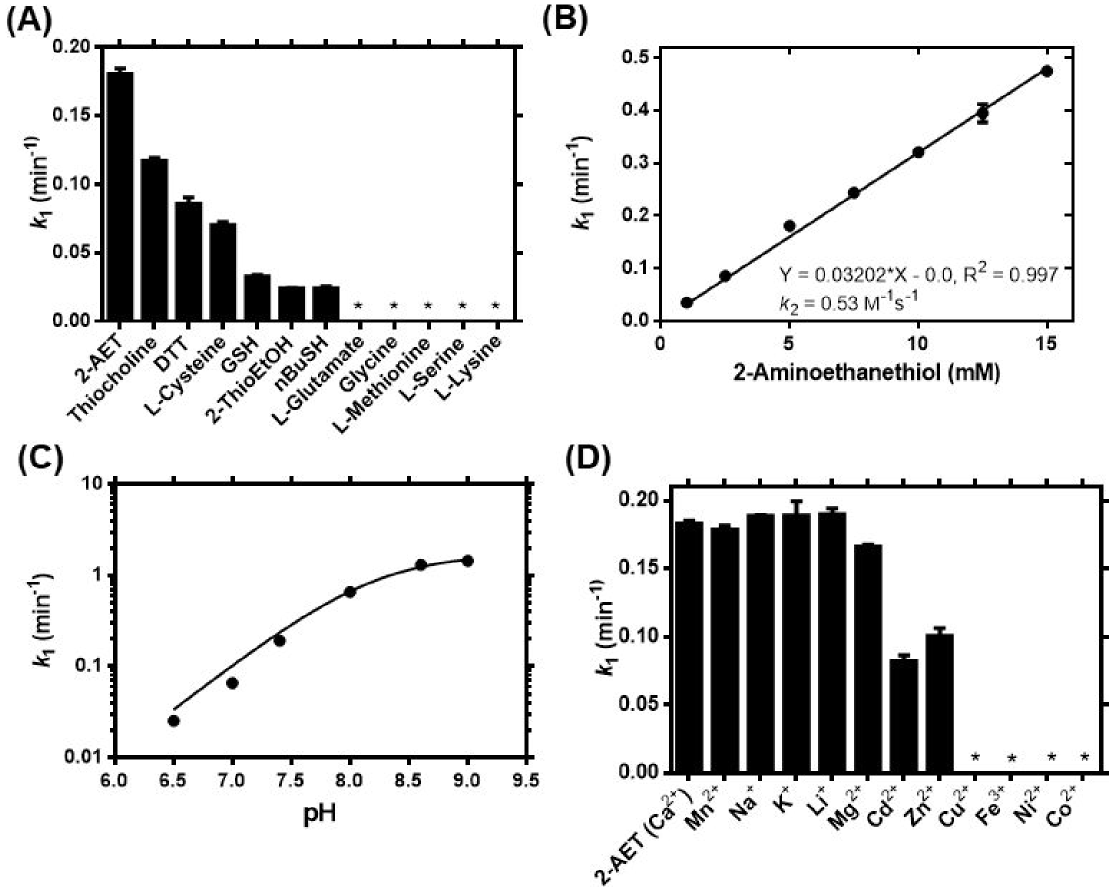

Figure S3. Kinetic studies of reactions between **asym6-6FAM** and various reactants in order to determine the common reaction mechanism of **asym6-6FAM** with thiols. (A) Reactions of **asym6-6FAM** with twelve reactants were conducted to determine values of the pseudo-first-order rate constant, k_1, for each reaction. The thiol-containing reactants were 2-aminoethanethiol (2-AET), thiocholine, DL-dithiothreitol (DTT), L-cysteine, glutathione (GSH), 2-mercaptoethanol (2-ThioEtOH), and 1-butanethiol (nBuSH). (B) The second-order reaction mechanism of **asym6-6FAM** with thiols was confirmed by pseudo-first-order kinetic analysis of reactions between **asym6-6FAM** and different [2-AET], which facilitated calculation of the corresponding second-order rate constant k_2, 0.53 M^{-1} s^{-1}. (C) pH titration studies of the 2-AET-**asym6-6FAM** reaction to provide evidence for a universal mechanism of general base catalysis in reactions of **asym6-6FAM** with thiols. (D) Effect of metal ions on the thiol-dependent, fluorogenic reactions of **asym6-6FAM** in the presence of 2-AET. Each reaction determined the effects of a metal ion (1 mM) on the pseudo-first-order reaction of **asym6-6FAM** (0.15 μM) with 5 mM of 2-AET in phosphate buffer (PB; 100 mM, pH 7.4). The k_1 values were similarly obtained from a method described in Experimental (SI) and in the legend of Figure S4. The asterisk symbol (*) indicates that the change in 6-FAM fluorescence in reactions was so minuscule that the values of averaged k_1 and standard deviation (the error bar) from

experiments performed in quadruplicate could not be determined. Moreover, some error bars were too small to be visible in the figures, even for those reactions with detectable fluorescence.

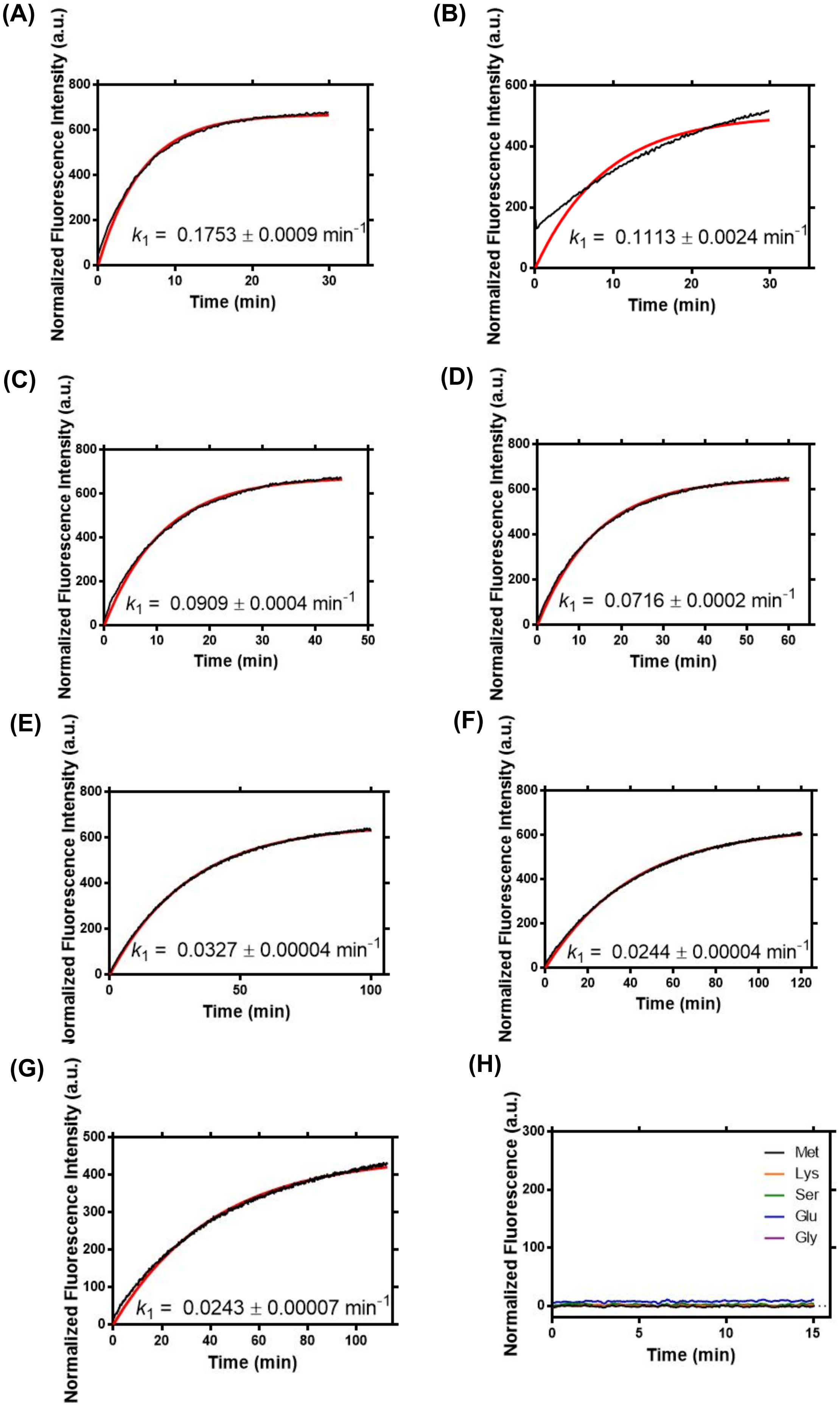

(A)
Normalized Fluorescence Intensity (a.u.)
Time (min)
k1 = 0.1753 ± 0.0009 min-1
(B)
Normalized Fluorescence Intensity (a.u.)
Time (min)
k1 = 0.1113 ± 0.0024 min-1
(C)
Normalized Fluorescence Intensity (a.u.)
Time (min)
k1 = 0.0909 ± 0.0004 min-1
(D)
Normalized Fluorescence Intensity (a.u.)
Time (min)
k1 = 0.0716 ± 0.0002 min-1
(E)
Normalized Fluorescence Intensity (a.u.)
Time (min)
k1 = 0.0327 ± 0.00004 min-1
(F)
Normalized Fluorescence Intensity (a.u.)
Time (min)
k1 = 0.0244 ± 0.00004 min-1
(G)
Normalized Fluorescence Intensity (a.u.)
Time (min)
k1 = 0.0243 ± 0.00007 min-1
(H)
Normalized Fluorescence (a.u.)
Time (min)
Met
Lys
Ser
Glu
Gly

Figure S4. Representative pseudo-first-order reactions of **asym6-6FAM** (0.15 μM) with 5 mM of (A) 2-aminoethanethiol (2-AET), (B) thiocholine, (C) DL-dithiothreitol (DTT), (D) L-cysteine, (E) glutathione (GSH), (F) 2-mercaptoethanol (2-ThioEtOH), (G) 1-butanethiol (nBuSH), or (H) five non-thiol amino acids (L-methionine, L-lysine, L-serine, L-glutamate, and glycine) in phosphate buffer (PB; 100 mM, pH 7.4). Progress of each reaction was monitored by measuring fluorescence emission at 515 nm at specific time intervals. Data of normalized 6-FAM fluorescence intensity (a.u.) *vs.* time were fitted to a single-exponential equation for first-order kinetics $F(t) = F_0 + F_{max}(1 - e^{-k_1 t})$ [$F(t)$, 6-FAM fluorescence at a specific time point t] to provide the values of first-order rate constant k_1 (GraphPad, La Jolla, CA, USA) illustrated in the graphs. In Panels S4A-S4G, the black traces indicate spectrometrically measured fluorescence changes in the reaction time courses, and the red curves show the results calculated by the single-exponential equation. The normalized fluorescence intensity data at 515 nm were acquired by subtracting a background fluorescence of **asym6-6FAM** from the original fluorescence intensity measurements. Kinetic experiments were performed in quadruplicate to determine k_1 for each reaction; the resulting k_1 values were the mean ± SD of the four experiments and were subsequently used to plot Figure S3A in SI.

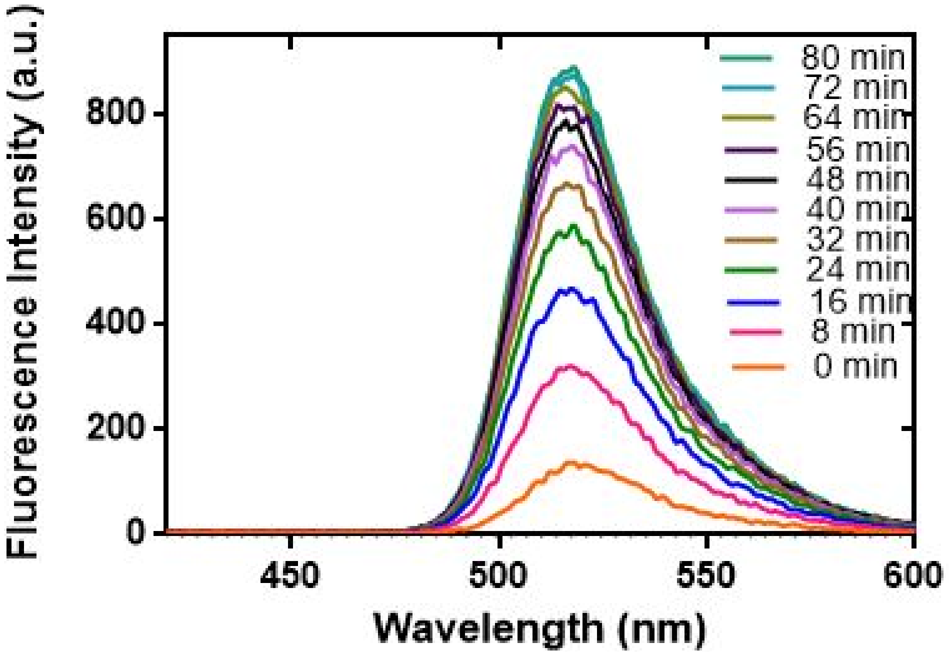

Figure S5. The contact quenching and release of the intrinsic 6-FAM fluorescence from the glutathione (GSH)-**asym6-6FAM** reaction as determined by fluorometric studies. The time-dependent measurements of the 6-FAM fluorescence were recorded from a reaction of **asym6-6FAM** (0.5 μM) with GSH (5 mM) in phosphate buffer (PB; 100 mM, pH 7.4).

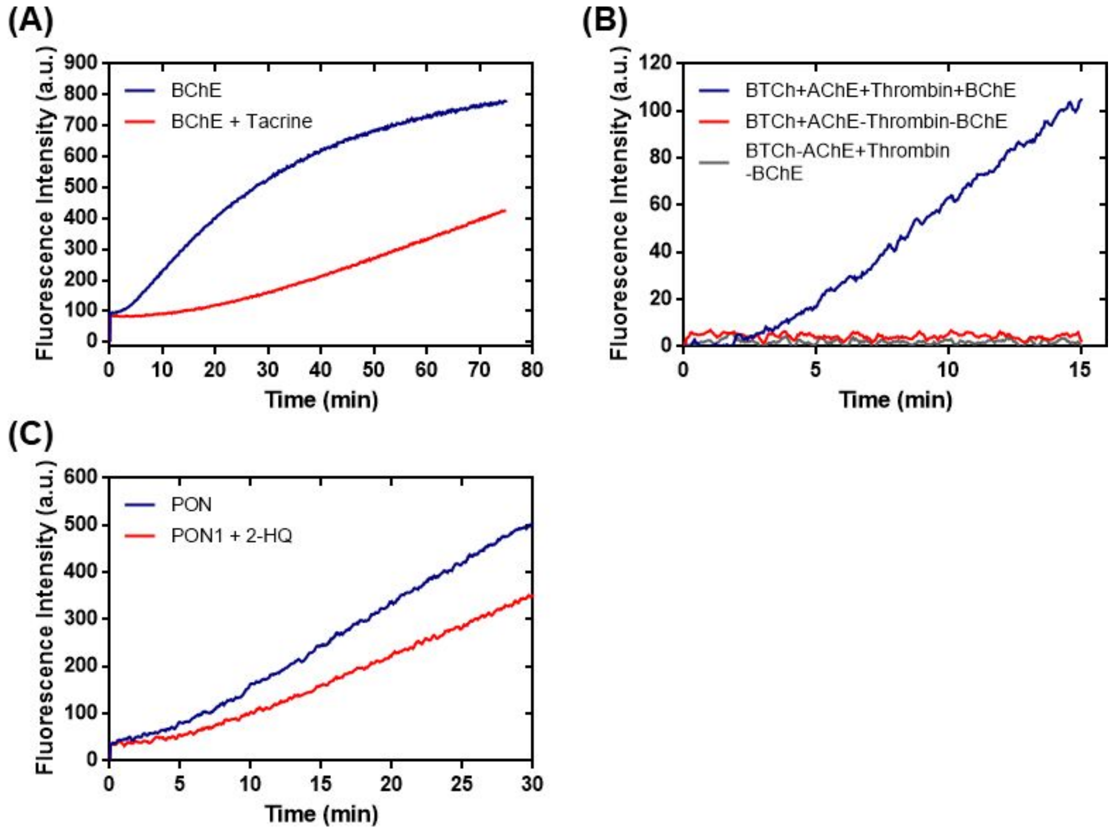

Figure S6. Emission of the 6-FAM fluorescence from **asym6-6FAM** critically depends on catalysis of BChE and PON1 as demonstrated by specific and competitive inhibition of tacrine and 2-hydroxyquinoline (2-HQ), and by inability of acetylcholinesterase (AChE) and the serum protease thrombin to affect the structural integrity of **asym6-6FAM** and to release the 6-FAM fluorescence from **asym6-6FAM**. (A) Specific and competitive inhibition of tacrine on the BChE-**asym6-6FAM** reaction. Time-course kinetics of tacrine inhibition of BChE (136.7 U L^{-1}; Merck Ltd., Taiwan) catalysis were obtained from reactions of BChE, *S*-butyrylthiocholine (BTCh, 250 μM), **asym6-6FAM** and tacrine (0.2 μM) in phosphate buffer (PB; 100 mM, pH 7.4) at 37˚C by following the 6-FAM fluorescence in the **asym6-6FAM**-based assay. The same BChE reaction, but without the presence of tacrine, was the positive control in the study. (B) Emission of the 6-FAM fluorescence from **asym6-6FAM** not relevant to structural change of **asym6-6FAM** contributed by the catalytic activities of AChE and/or thrombin. Time-course kinetics of **asym6-6FAM**-BTCh (250 μM) reactions in the presence or absence of BChE (136.7 U L^{-1}), AChE (666.7 U L^{-1}; Merck Ltd., Taiwan) or thrombin (666.7 U L^{-1}; Merck Ltd., Taiwan) in PB at 37˚C were studied by following 6-FAM fluorescence measurements. (C) 2-HQ specifically and competitively inhibits the PON1-**asym6-6FAM** reaction. A PON1-**asym6-6FAM**-2-HQ reaction system, which contained **asym6-6FAM** (0.7 μM in DMSO, 2.2%), recombinant PON1 (rePON1; 143.0 U L^{-1}, 22.6% glycerol), 2-HQ (30 μM) and 5-thiobutyl butyrolactone (TBBL; 10 mM in acetonitrile, 2%) in Tris

buffer (50 mM of Tris, 1 mM of Ca^{2+}, pH 8.0) was used to investigate 2-HQ inhibition at 25 ˚C by monitoring the change of the 6-FAM fluorescence during the course of the reaction. The same PON1 reaction, but in the absence of 2-HQ, was the positive control. The normalized fluorescence intensity at 515 nm was calculated by subtracting background fluorescence of **asym6-6FAM** at the same wavelength from the original fluorescence intensity data.

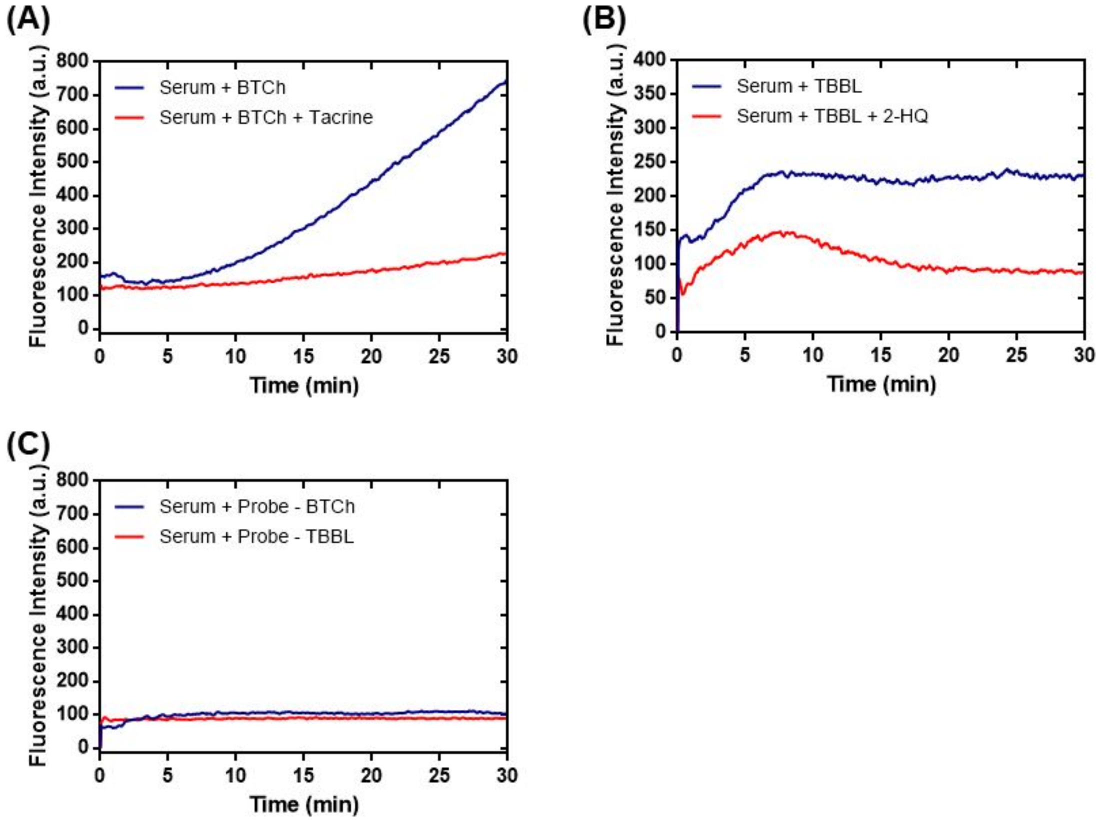

Figure S7. Serum biothiols and serum enzymes other than BChE and PON1 do not interfere with measuring either BChE or PON1 lactonase activity in the **asym6-6FAM**-based assays, and interactions between serum proteins and **asym6-6FAM** do not contribute to increases of the 6-FAM fluorescence in the **asym6-6FAM**-based assays. (A) Specific and competitive inhibition of tacrine in the serum-*S*-butyrylthiocholine (BTCh)-**asym6-6FAM** reaction. Time-course kinetics of tacrine inhibition on BChE catalysis in serum (100-fold dilution) was obtained from the reaction of the diluted serum, BTCh (1 mM), **asym6-6FAM** (0.3 μM, 0.6% DMSO) and tacrine (0.2 μM) in phosphate buffer (PB; 100 mM, pH 7.4) at 37˚C by following changes of 6-FAM fluorescence levels in the **asym6-6FAM**-based assay. The same serum-BTCh-**asym6-6FAM** reaction, but without the presence of tacrine, was the positive control. (B) 2-Hydroxyquinoline (2-HQ) specifically and

competitively inhibits PON1 catalysis in the serum-5-thiobutyl butyrolactone (TBBL)-**asym6-6FAM** reaction. A serum-TBBL-**asym6-6FAM**-2-HQ reaction system, which contained **asym6-6FAM** (0.7 μM in DMSO, 2.2%), serum (750-fold dilution), 2-HQ (30 μM) and TBBL (10 mM in acetonitrile, 2%) in Tris buffer (50 mM of Tris, 1 mM of Ca^{2+}, pH 8.0), was used to determine 2-HQ inhibition at 25°C by monitoring the change in the 6-FAM fluorescence during the course of the reaction. The same serum-TBBL-**asym6-6FAM** reaction, but in the absence of 2-HQ, was the positive control in the study. (C) Effects of interactions between serum proteins and **asym6-6FAM** in the **asym6-6FAM**-based assays. The blue curve represents time-course kinetics of the serum-**asym6-6FAM** reaction in the absence of BTCh, a specific substrate of BChE catalysis in serum. This reaction contained serum (100-fold dilution) and **asym6-6FAM** (0.3 μM, 0.6% DMSO) in phosphate buffer (PB; 100 mM, pH 7.4) at 37˚C and was monitored by following the change of the 6-FAM fluorescence in the **asym6-6FAM**-based assay. Similarly, the red curve displays the time-course kinetics of the serum-**asym6-6FAM** reaction in the absence of TBBL, which is a specific substrate of PON1 lactonase catalysis in serum. The reaction comprised serum (750-fold dilution) and **asym6-6FAM** (0.7 μM in DMSO, 2.2%) in the Tris buffer and measured changes in the 6-FAM fluorescence during the reaction using the fluorescence spectrometer. The normalized fluorescence intensity at 515 nm was calculated by subtracting background fluorescence of **asym6-6FAM** at the same wavelength from the original fluorescence intensity data.

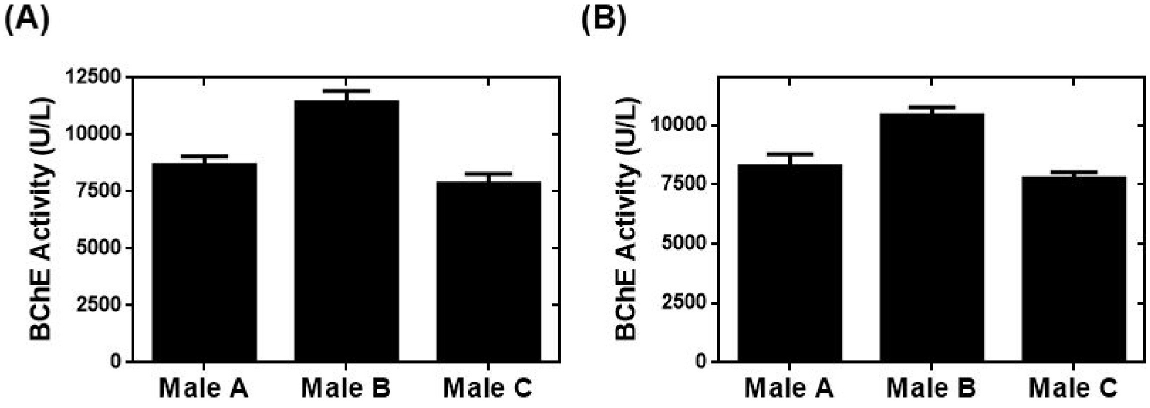

Figure S8. The standard Ellman's assay[7] for determining BChE activity in the serum samples from the same three healthy males described in Figures 3 and 5 (A) by the UV-Vis spectrophotometer-based method or (B) by the high-throughput, microplate reader-based method.

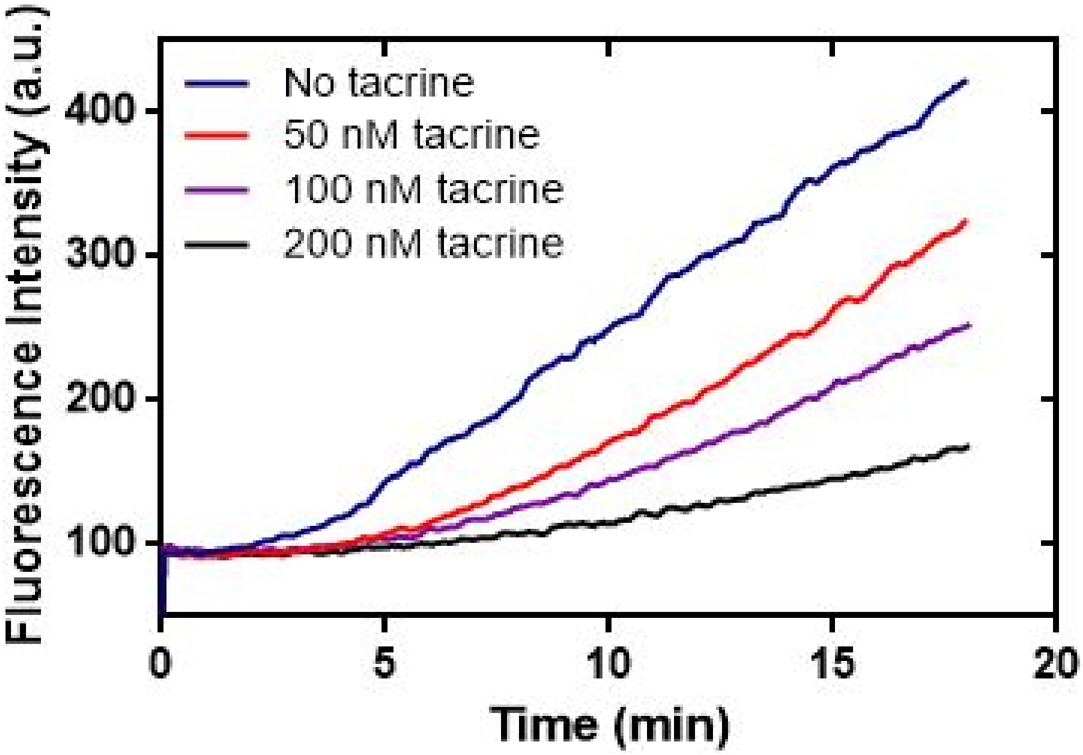

Figure S9. Representative curves of tacrine inhibition of BChE catalysis determined by the fluorescence assay based on **asym6-6FAM**. Time-course kinetic analysis of tacrine inhibition of BChE (136.7 U L^{-1}) catalysis was performed in the presence of **asym6-6FAM** (0.3 μM), BTCh (300 μM) and tacrine (0, 50, 100 or 200 nM) in phosphate buffer (PB; 100 mM, pH 7.4) at 37 °C. Each specific BChE catalysis reaction was performed in triplicate to acquire v_i as the mean ± SD and to plot Figure 4B in the main text.

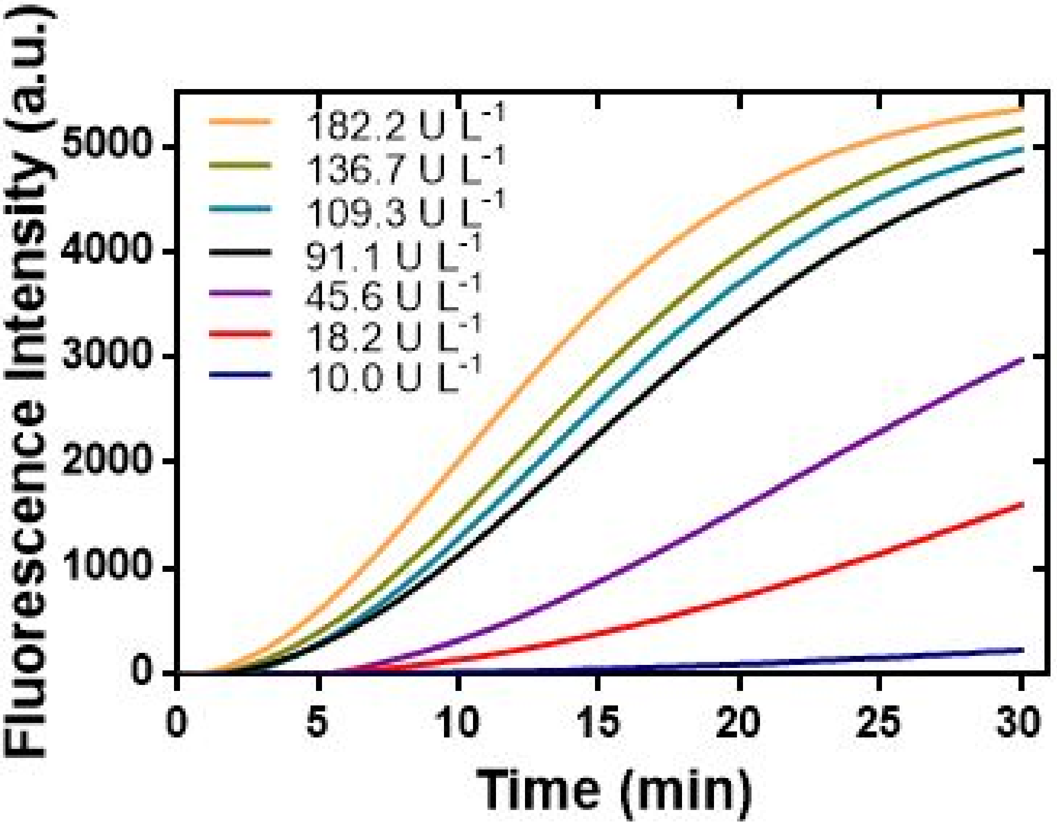

Figure S10. Representative curves of high-throughput measurements of BChE activity in the **asym6-6FAM**-based fluorescence turn-on assay. High-throughput kinetic studies of BChE (10-182.2 U L^{-1}) catalysis detected changes of the 6-FAM fluorescence during time course reactions containing **asym6-6FAM** (9 μM) and BTCh (1 mM) in phosphate buffer (PB; 100 mM, pH 7.4) at 37˚C. Seven representative BChE catalytic reactions in which each reaction employed a different level of BChE activity are shown. Each specific BChE high-throughput analysis was performed in triplicate to acquire v_i as the mean ± SD and to plot Figure 5A in the main text.

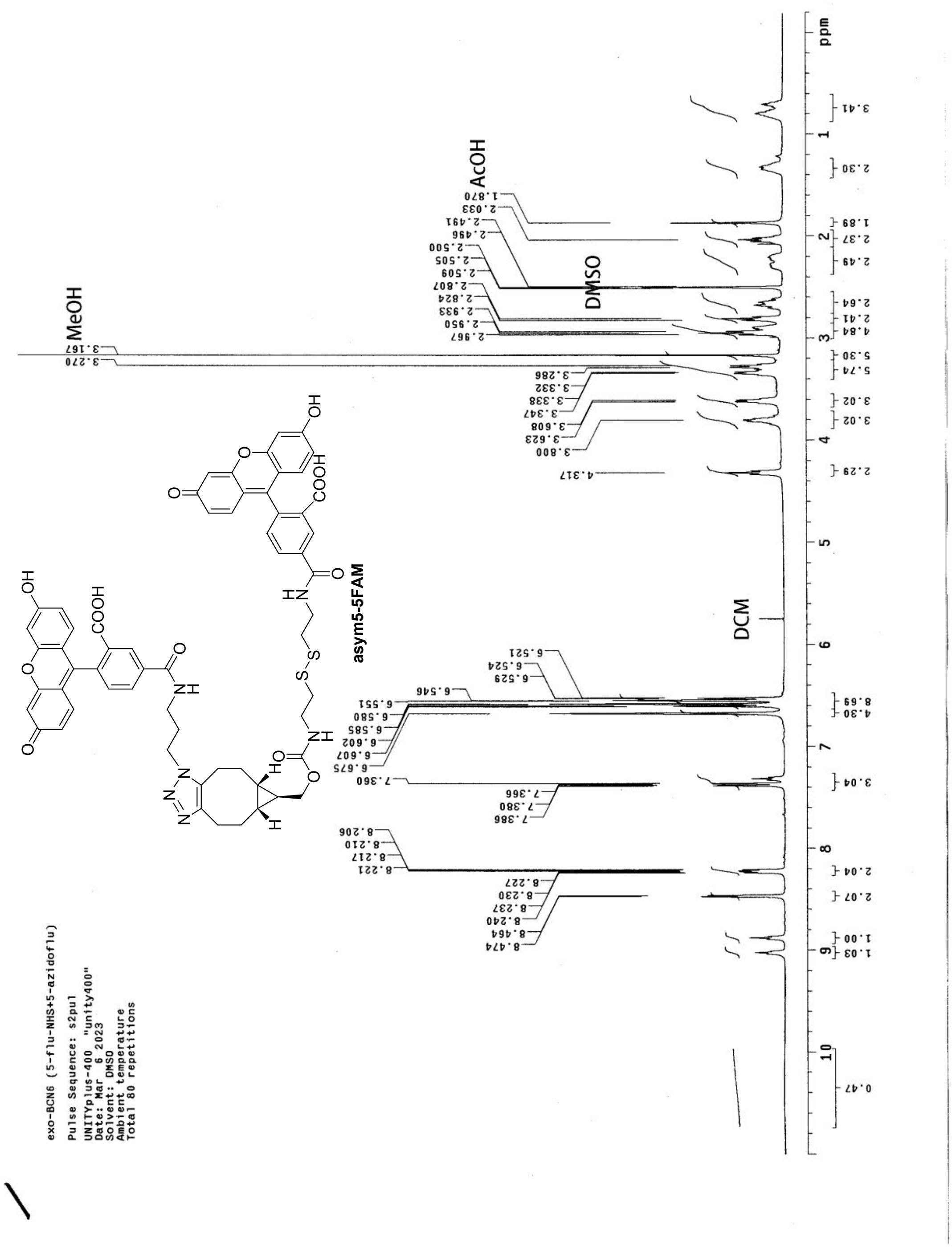

Figure S11. The ^{1}H NMR spectrum of **asym5-5FAM**.

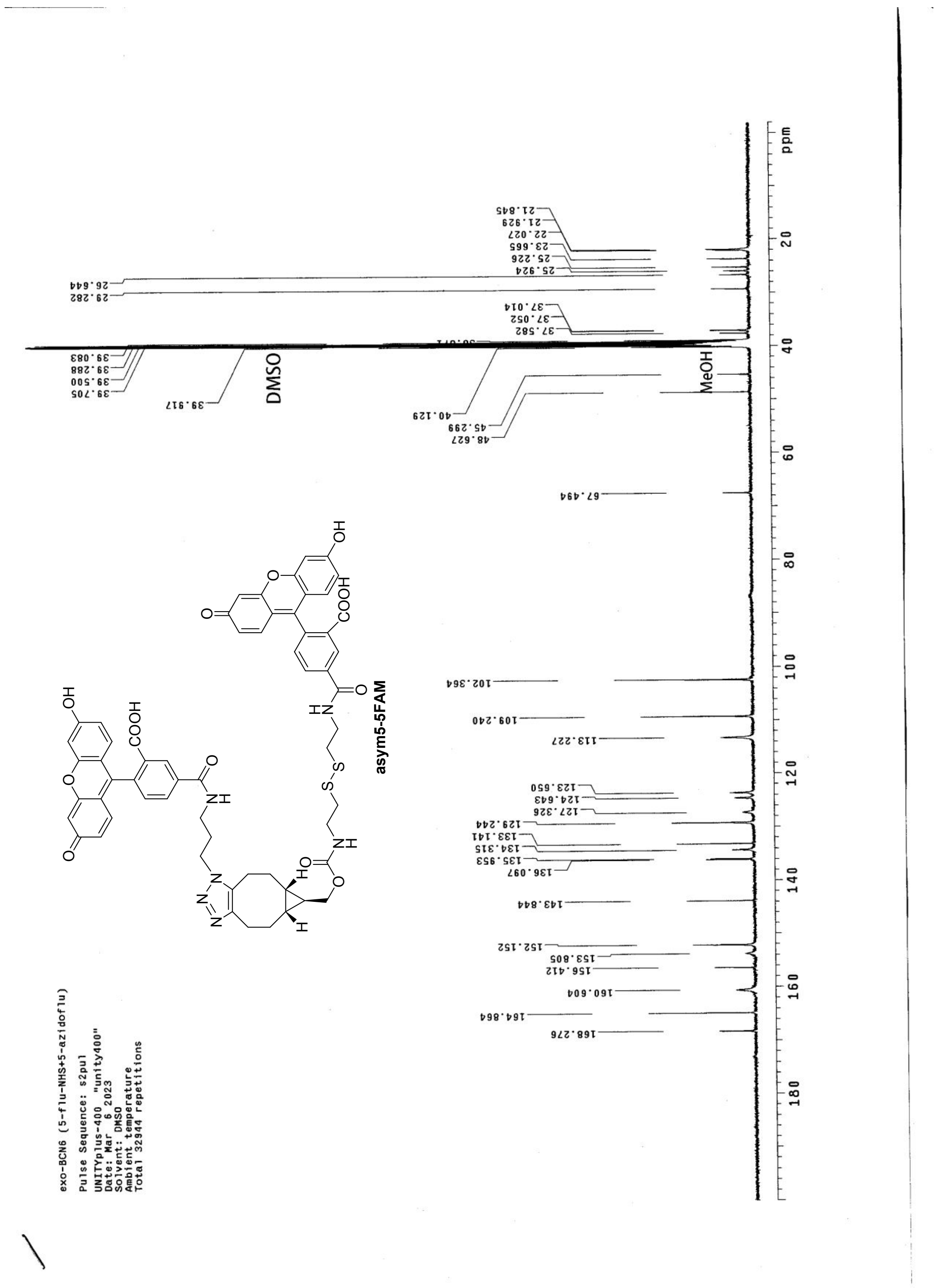

Figure S12. The ^{13}C NMR spectrum of **asym5-5FAM**.

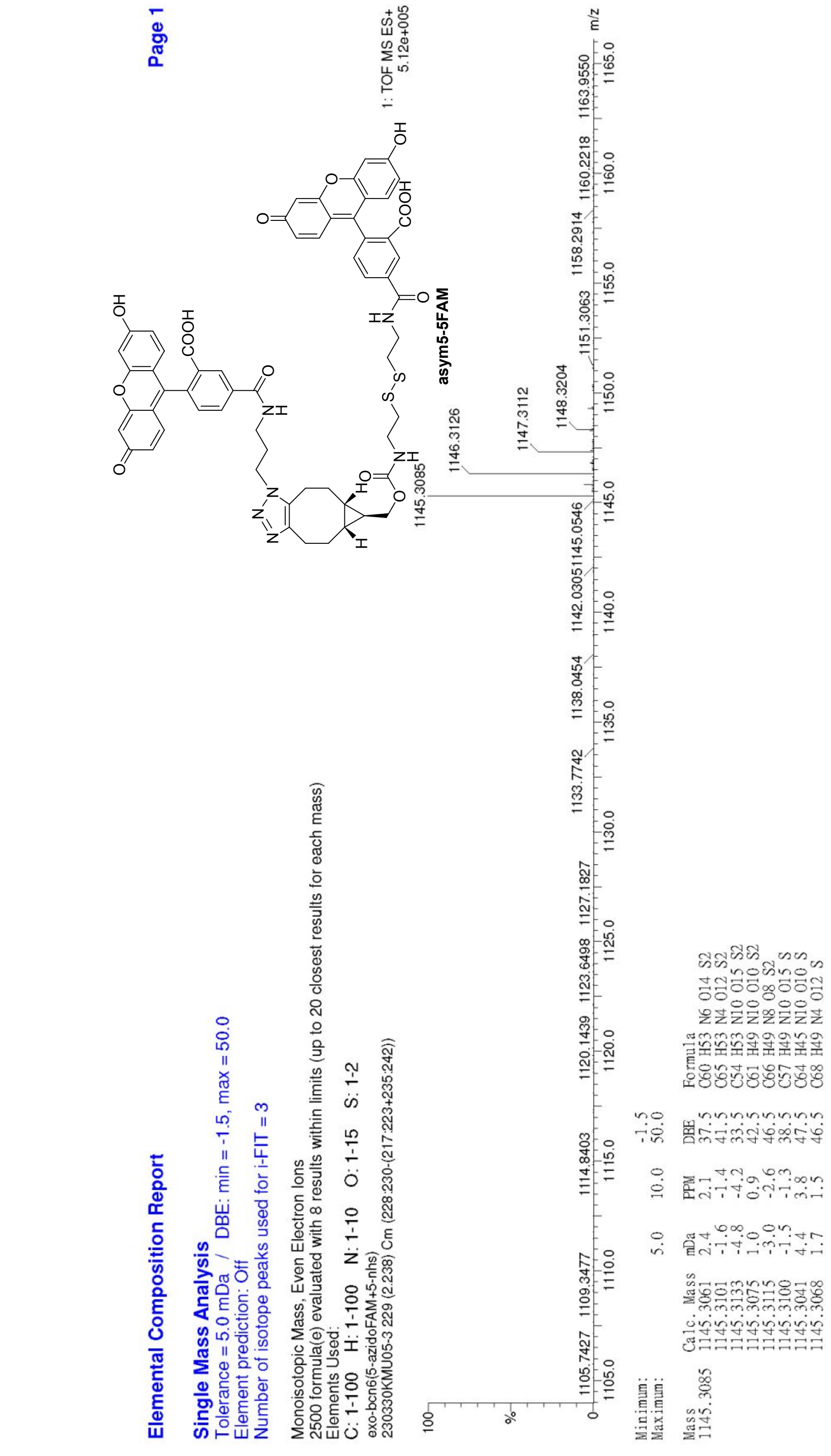

Figure S13. The HRMS spectrum of **asym5-5FAM**.

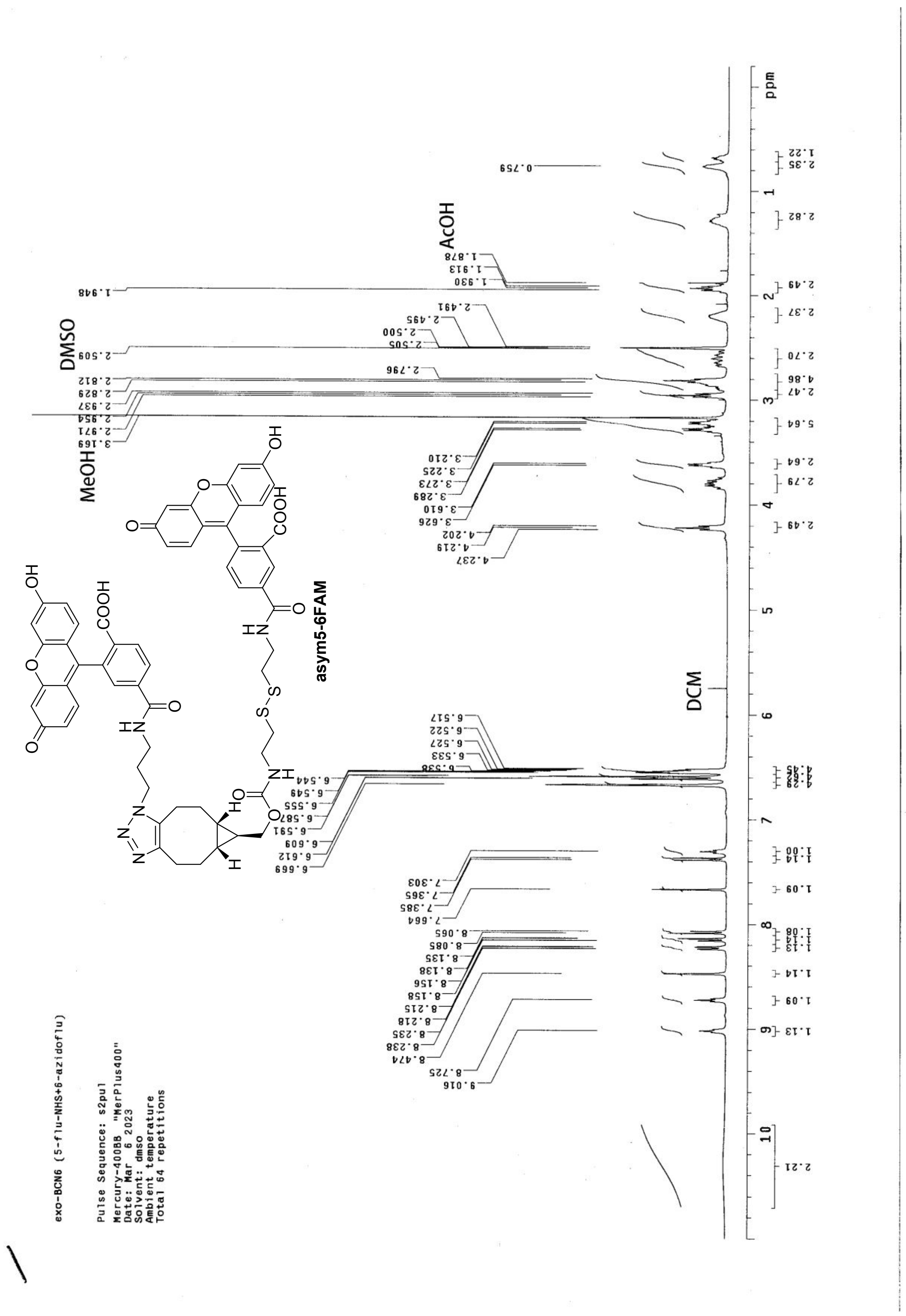

Figure S14. The ^{1}H NMR spectrum of **asym5-6FAM**.

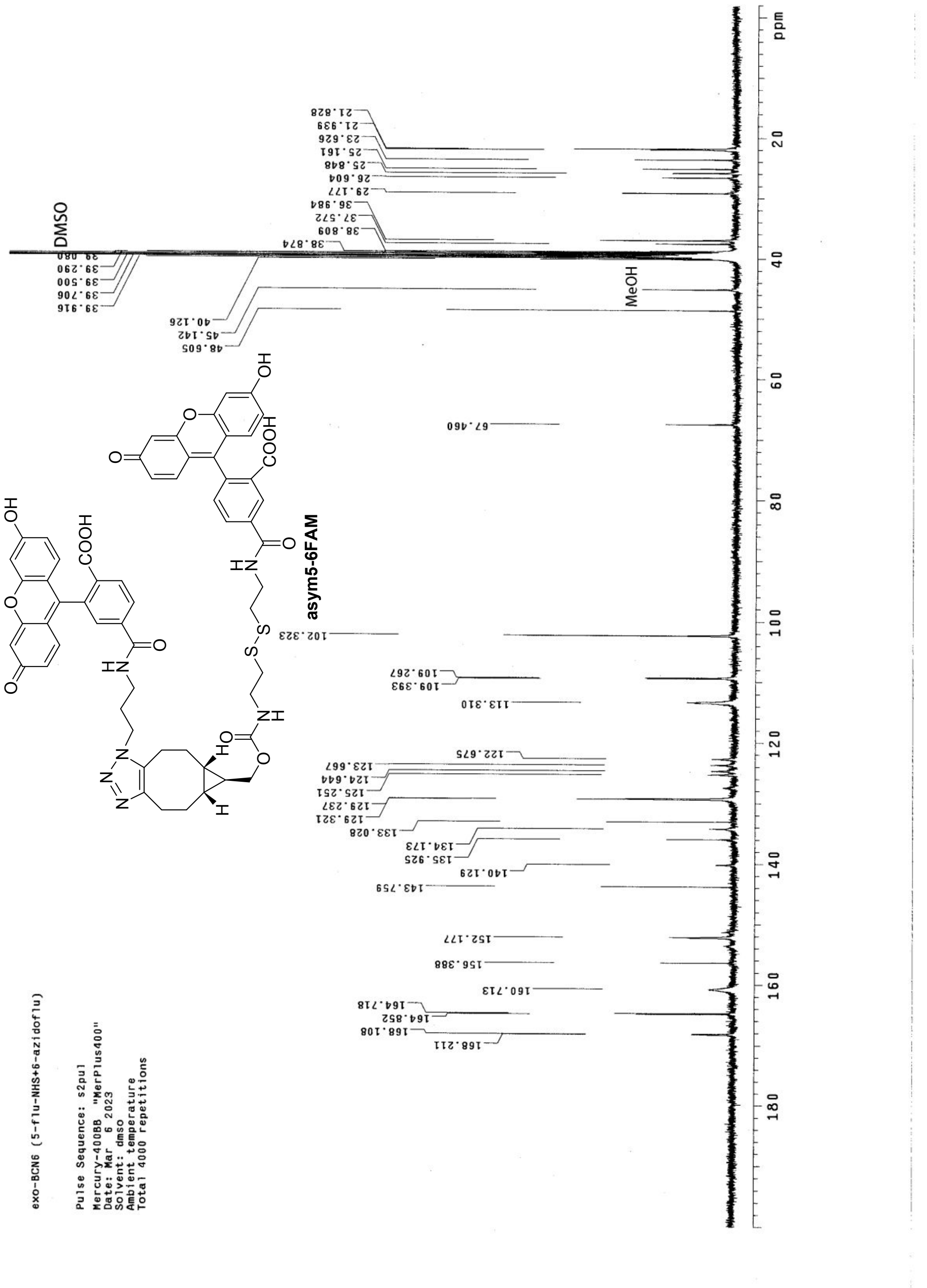

Figure S15. The ^{13}C NMR spectrum of **asym5-6FAM**.

Elemental Composition Report

Page 1

Single Mass Analysis

Tolerance = 5.0 mDa / DBE: min = -1.5, max = 50.0
Element prediction: Off
Number of isotope peaks used for i-FIT = 3

Monoisotopic Mass, Even Electron Ions
2500 formula(e) evaluated with 7 results within limits (up to 20 closest results for each mass)
Elements Used:
C: 1-100 H: 1-100 N: 1-10 O: 1-15 S: 1-2
exo-bcn6(5-azidoFAM+6-nhs)
230330KMU06 235 (2.309) Cm (235:237-(224:230+244:252))

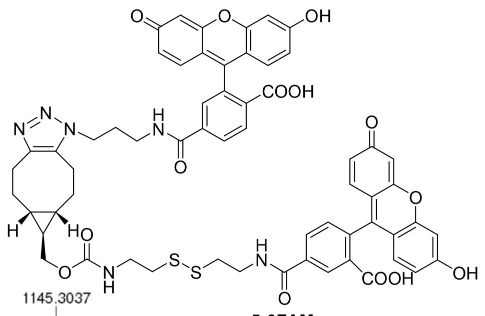

asym5-6FAM

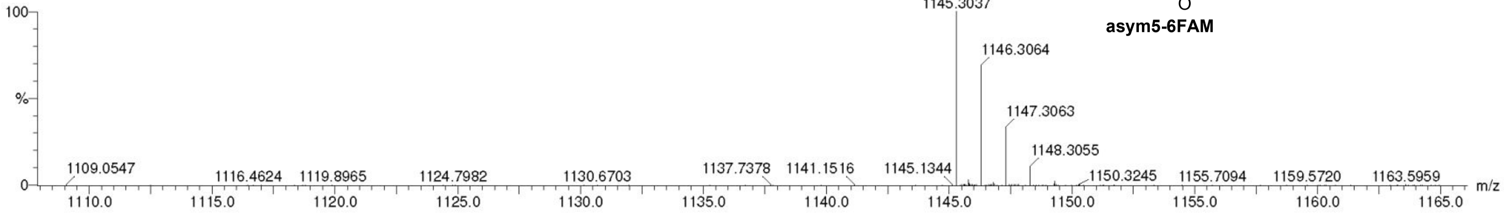

		mDa	PPM	DBE	
Minimum:				-1.5	
Maximum:		5.0	10.0	50.0	

Mass	Calc. Mass	mDa	PPM	DBE	Formula
1145.3037	1145.3061	-2.4	-2.1	37.5	C60 H53 N6 O14 S2
	1145.3027	1.0	0.9	42.5	C63 H49 N6 O14 S
	1145.2989	4.8	4.2	41.5	C66 H53 N2 O13 S2
	1145.3068	-3.1	-2.7	46.5	C68 H49 N4 O12 S
	1145.3041	-0.4	-0.3	47.5	C64 H45 N10 O10 S
	1145.3075	-3.8	-3.3	42.5	C61 H49 N10 O10 S2
	1145.3002	3.5	3.1	46.5	C67 H49 N6 O9 S2

Figure S16. The HRMS spectrum of **asym5-6FAM**.

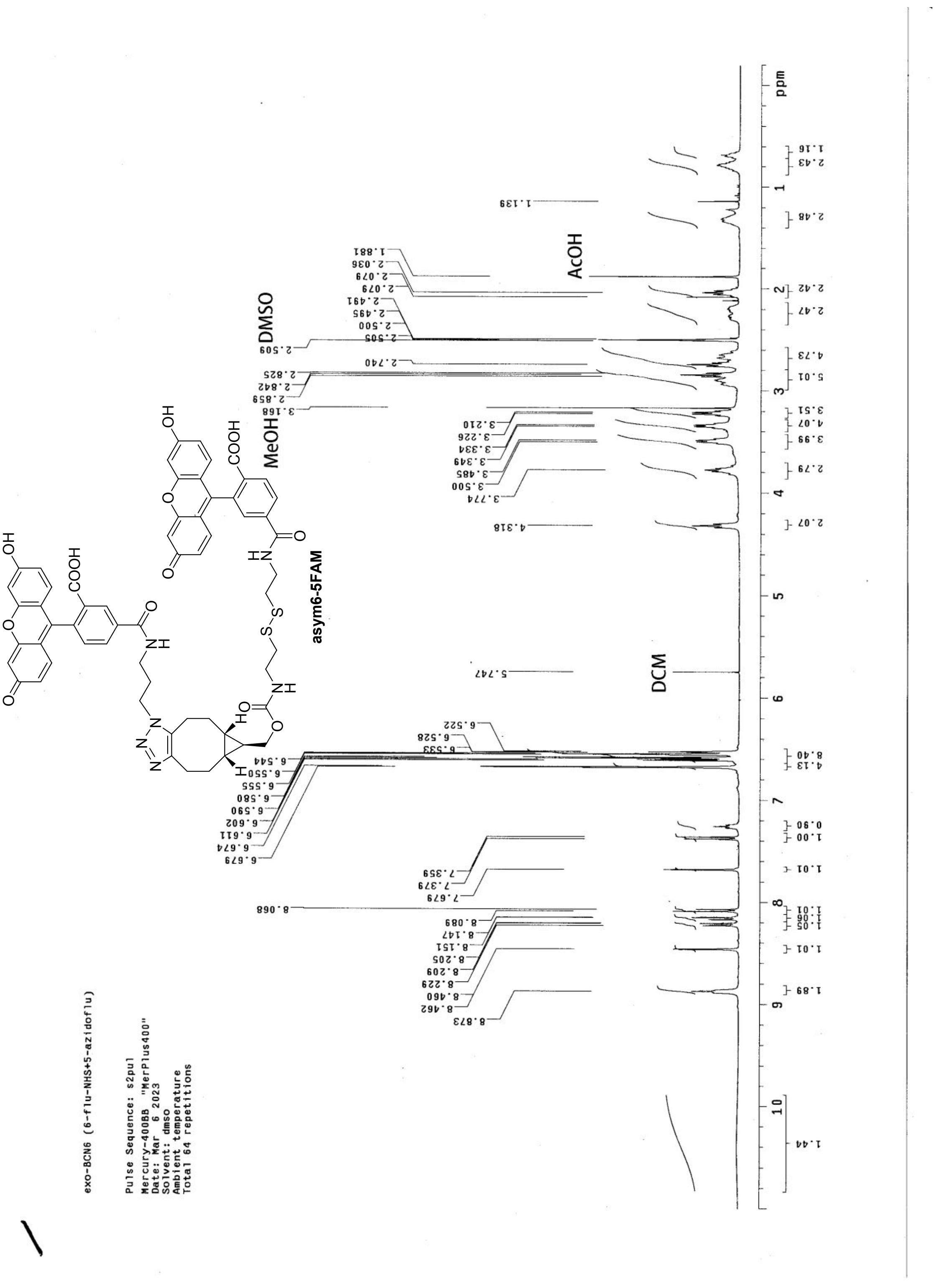

Figure S17. The ^{1}H NMR spectrum of **asym6-5FAM**.

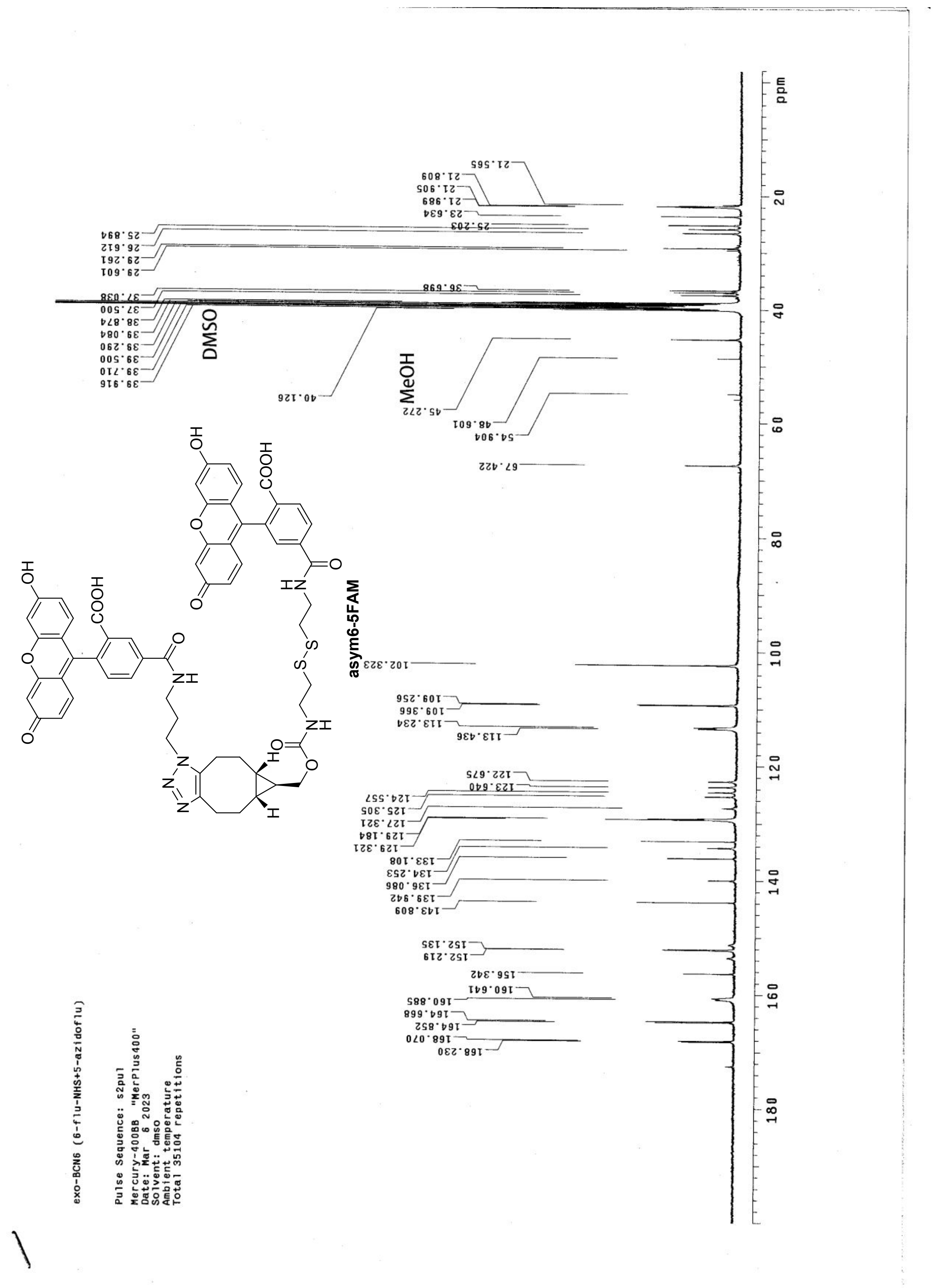

Figure S18. The ^{13}C NMR spectrum of **asym6-5FAM**.

Elemental Composition Report

Page 1

Single Mass Analysis

Tolerance = 5.0 mDa / DBE: min = -1.5, max = 50.0
Element prediction: Off
Number of isotope peaks used for i-FIT = 3

Monoisotopic Mass, Even Electron Ions
2500 formula(e) evaluated with 6 results within limits (up to 20 closest results for each mass)
Elements Used:
C: 1-100 H: 1-100 N: 1-10 O: 1-15 S: 1-2
exo-bcn6(6-azidoFAM+5-nhs)
230330KMU07-3 227 (2.220) Cm (226:228-(217:221+235:239))

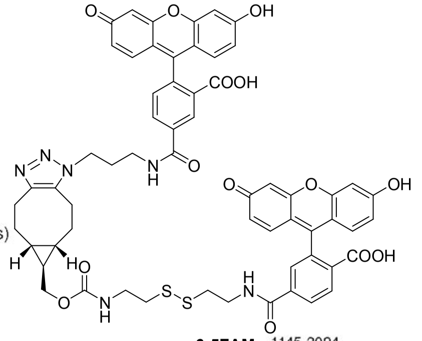

asym6-5FAM

1: TOF MS ES+
5.04e+005

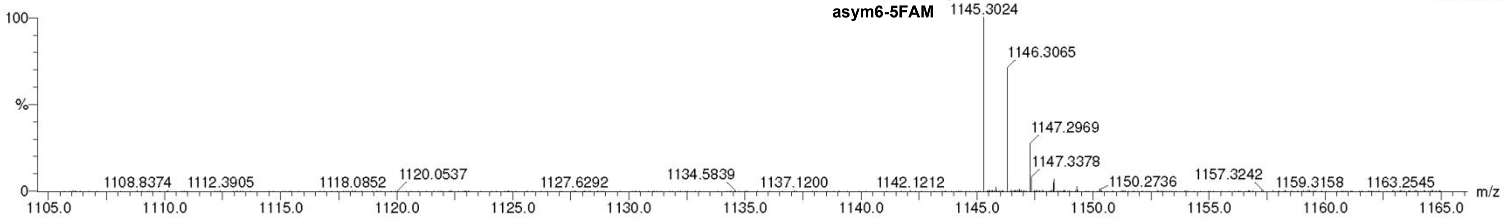

		mDa	PPM	DBE	
Minimum:				-1.5	
Maximum:		5.0	10.0	50.0	

Mass	Calc. Mass	mDa	PPM	DBE	Formula
1145.3024	1145.3061	-3.7	-3.2	37.5	C60 H53 N6 O14 S2
	1145.3027	-0.3	-0.3	42.5	C63 H49 N6 O14 S
	1145.2989	3.5	3.1	41.5	C66 H53 N2 O13 S2
	1145.3068	-4.4	-3.8	46.5	C68 H49 N4 O12 S
	1145.3041	-1.7	-1.5	47.5	C64 H45 N10 O10 S
	1145.3002	2.2	1.9	46.5	C67 H49 N6 O9 S2

Figure S19. The HRMS spectrum of **asym6-5FAM**.

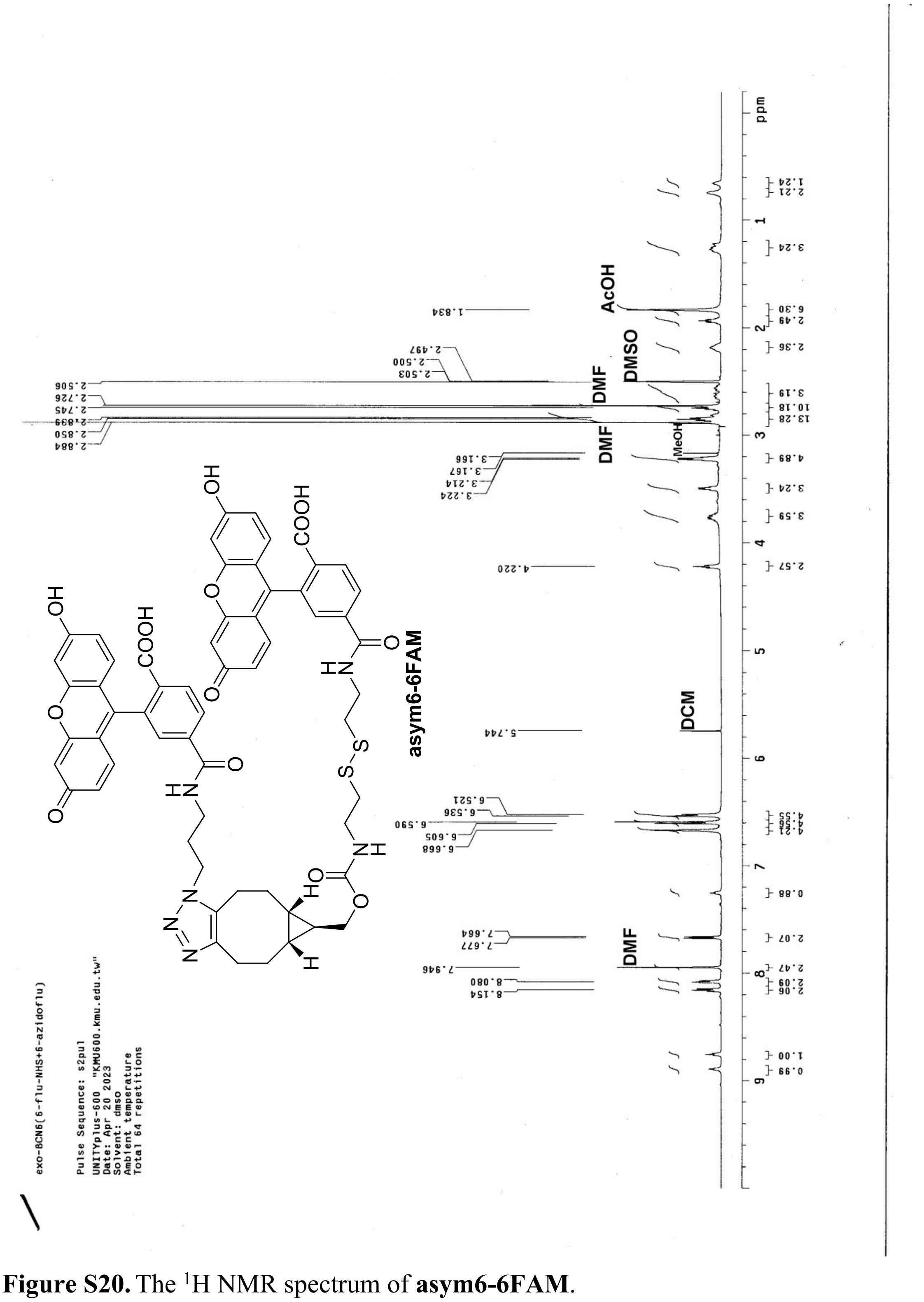

Figure S20. The ^{1}H NMR spectrum of **asym6-6FAM**.

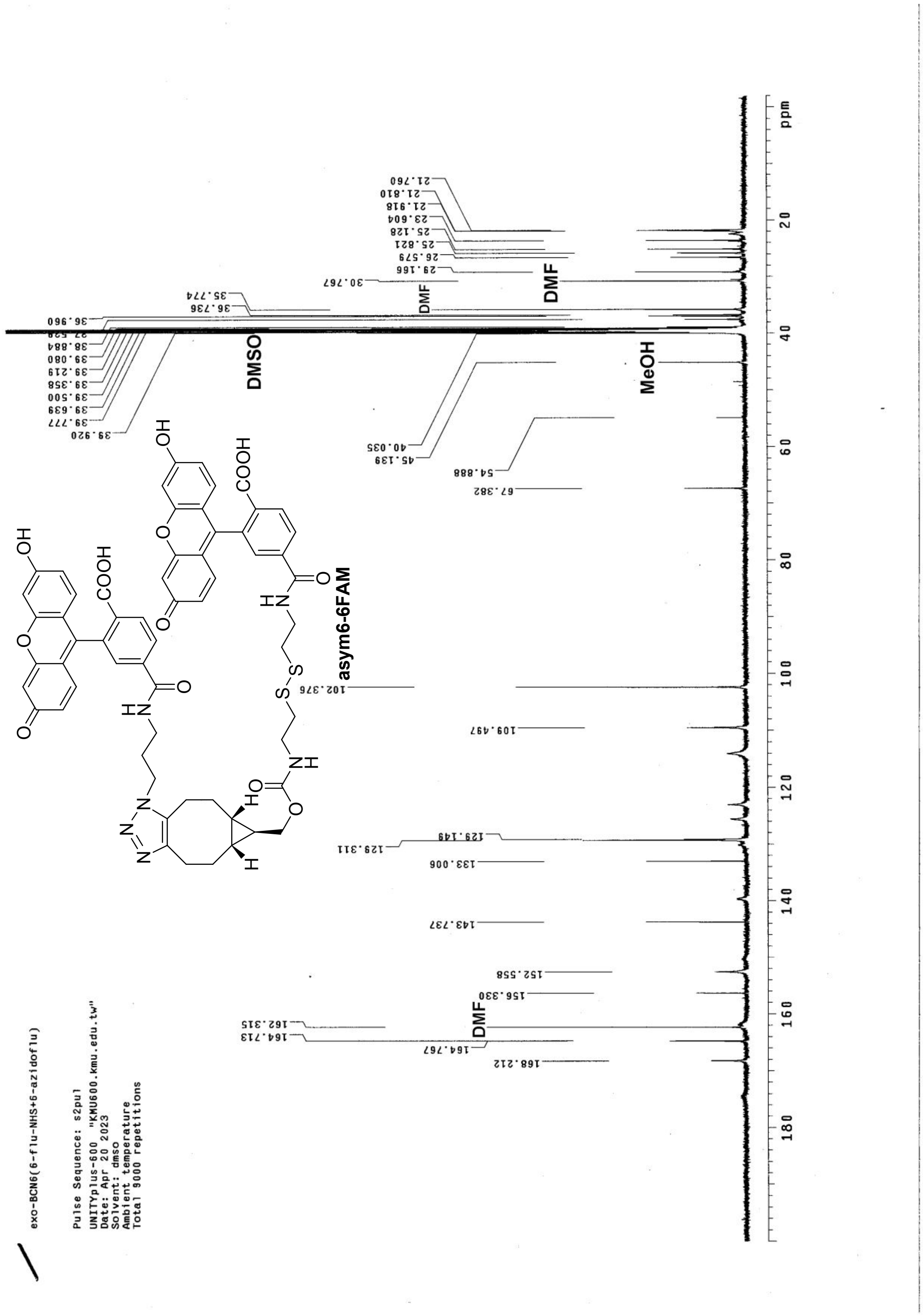

Figure S21. The ^{13}C NMR spectrum of **asym6-6FAM**.

Elemental Composition Report

Page 1

Single Mass Analysis

Tolerance = 10.0 mDa / DBE: min = -1.5, max = 50.0
Element prediction: Off
Number of isotope peaks used for i-FIT = 3

Monoisotopic Mass, Even Electron Ions
2500 formula(e) evaluated with 15 results within limits (up to 20 closest results for each mass)
Elements Used:
C: 1-100 H: 1-100 N: 1-10 O: 1-15 S: 1-2
exo-bcn6(6-azidoFAM+6-nhs)
230330KMU08-4 226 (2.212) Cm (225:227-(217:221+234:236))

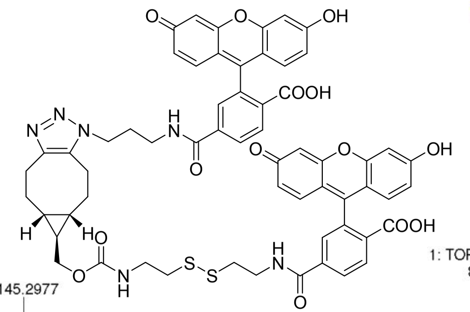

asym6-6FAM

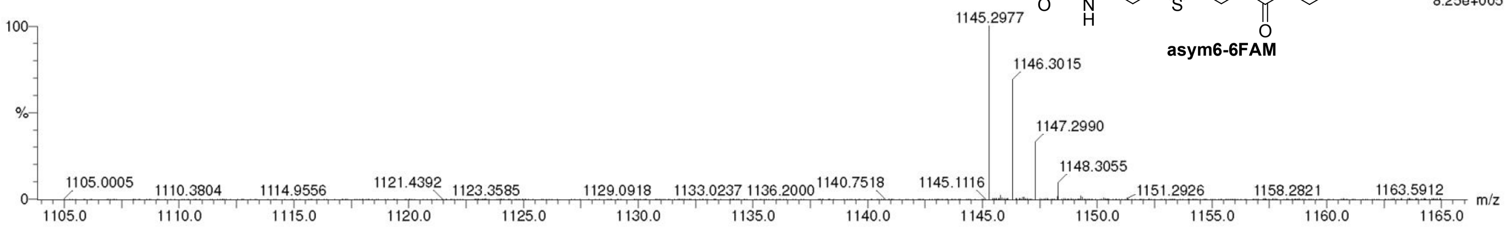

Minimum:				-1.5	
Maximum:		10.0	10.0	50.0	

Mass	Calc. Mass	mDa	PPM	DBE	Formula
1145.2977	1145.3061	-8.4	-7.3	37.5	C60 H53 N6 O14 S2
	1145.2989	-1.2	-1.0	41.5	C66 H53 N2 O13 S2
	1145.2949	2.8	2.4	37.5	C61 H53 N4 O15 S2
	1145.3002	-2.5	-2.2	46.5	C67 H49 N6 O9 S2
	1145.3068	-9.1	-7.9	46.5	C68 H49 N4 O12 S
	1145.2890	8.7	7.6	46.5	C68 H49 N4 O10 S2
	1145.2955	2.2	1.9	46.5	C69 H49 N2 O13 S
	1145.2915	6.2	5.4	42.5	C64 H49 N4 O15 S
	1145.3027	-5.0	-4.4	42.5	C63 H49 N6 O14 S
	1145.2962	1.5	1.3	42.5	C62 H49 N8 O11 S2
	1145.3075	-9.8	-8.6	42.5	C61 H49 N10 O10 S2
	1145.2922	5.5	4.8	38.5	C57 H49 N10 O13 S2
	1145.2888	8.9	7.8	43.5	C60 H45 N10 O13 S
	1145.2929	4.8	4.2	47.5	C65 H45 N8 O11 S
	1145.3041	-6.4	-5.6	47.5	C64 H45 N10 O10 S

Figure S22. The HRMS spectrum of **asym6-6FAM**.

Table

Table S1. Contact quenching efficiencies of the four *bis*-FAM-containing chemical probes

Quenching Efficiency Determination

	Fluorescence Intensity Measurements			Φ_A Measurements	
Compounds	*Initial Fluorescence Intensity at 515 nm (a.u.)*[a]	*Maximal Fluorescence Intensity at 515 nm (a.u.)*	***Quenching Efficiency (%)***[b]	*Absolute Quantum Yield* (Φ_A)	***Quenching Efficiency (%)***[c]
**11**	N/A	N/A	**N/A**	85.3	**N/A**
**asym5-5FAM**	75	570	**87**	6.7	**92**
**asym5-6FAM**	136	782	**83**	9.2	**89**
**asym6-5FAM**	121	539	**78**	12.1	**86**
**asym6-6FAM**	110	893	**88**	4.6	**95**

[a]Initial fluorescence intensity was measured at 0 min.

[b]Quenching efficiency was calculated by subtracting the ratio of fluorescence intensity of λ_{max} at time 0 relative to the maximum intensity of λ_{max} expressed in % from 100%. λ_{max} was 515 nm in this study.

[c]Quenching efficiency was calculated by subtracting the ratio of Φ_A of a specific chemical probe relative to Φ_A of 5(6)-carboxyfluorescein (**11**) expressed in % from 100%. The excitation wavelength was 470 nm in this study.

Experimental

Reagent-grade chemicals were purchased from commercial vendors (Merck, Thermo Fisher, and TCI) and further purified as necessary. ^{1}H and ^{13}C NMR spectra were recorded using a Varian 200, 400, or 600 MHz spectrometer (Varian, Inc., Palo Alto, CA, USA) at Kaohsiung Medical University (KMU), Taiwan. NMR samples were prepared in $(CD_3)_2SO$, and the chemical shifts of ^{1}H and ^{13}C signals were reported in parts per million based on the internal standard of the deuterated solvent. ESI high-resolution mass spectra were acquired using micrOTOF-Q mass spectrometry (Bruker Taiwan Co Ltd., Taiwan) at the Department of Chemistry, National Sun Yat-Sen University, Taiwan. 5-Thiobutyl butyrolactone (TBBL), the specific substrate required for the measurement of paraoxonase 1 (PON1) lactonase activity, was synthesized according to a published method.[8] Synthesis of thiocholine, a thiol utilized in kinetic studies of pseudo-first-order reactions, was accomplished by following the method of Bernardes *et al*.[9]

Synthesis of the *mono-exo*-BCN (*exo*-bicyclo[6.1.0]nonyne)-containing and *bis*-carboxyfluorescein (FAM)-paired fluorescence turn-on chemical probes (Schemes S1-S3)

exo-BCN-6, 5-carboxyfluorescein (5-FAM) NHS ester (7), 6-carboxyfluorescein (6-FAM) NHS ester (8), exo-BCN-9, exo-BCN-10, 5-azido-FAM (14a) and 6-azido-FAM (14b). Synthesis of ***exo*-BCN-6**, 5-FAM NHS ester (**7**), 6-FAM-NHS ester (**8**), ***exo*-BCN-9** and ***exo*-BCN-10** was achieved by following previously reported methods (Schemes S1 and S3).[4] [3] 5-Azido-FAM (**14a**) and 6-azido-FAM (**14b**) were synthesized from **7** and **8**, respectively, by following an established method (Scheme S3).[5]

asym5-5FAM. A mixture of ***exo*-BCN-9** (30.0 mg, 0.044 mmol, 1 equiv.) and azido-6-carboxyfluorescein **14a** (22.1 mg, 0.048 mmol, 1.1 equiv) in 1,4-dioxane (2 mL) was heated under reflux for 6 h (Scheme S2). Upon cooling to rt, the reaction mixture was evaporated under reduced pressure to yield a solid residue, which was later resuspended in a limited volume of a DCM/acetone/AcOH (66:33:1, v/v/v) solution and loaded onto a silica gel (45-75 μm) column pre-equilibrated with the same eluent. The same DCM/acetone/AcOH solution was used to wash out impurities from the column in the dark. Additional compounds were eluted out of the column initially by a second DCM/acetone/AcOH (60:40:1, v/v/v) solution and finally by a third DCM/MeOH/AcOH (85:17:1, v/v/v) solution. The product-containing fractions were pooled and evaporated under reduced pressure to yield **asym5-5FAM** (39.4 mg , 0.034 mmol, 79%) as an orange solid. ^{1}H NMR (C_2D_6OS, 400 MHz) δ: 9.02 (t, 1H, J = 5.2 Hz), 8.88 (t, 1H, J = 5.2 Hz), 8.47 (d, 2H, J = 8 Hz), 8.22 (q, 2H, J = 6.8 Hz), 7.37 (d, 2H, J = 5.2 Hz), 7.31 (t, 1H, J = 6 Hz), 6.68 (s, 4H), 6.61-6.52 (m, 8H), 4.32 (t, 2H, J = 7 Hz), 3.81 (t, 2H, J = 8.5 Hz), 3.61 (d, 2H, J = 6 Hz), 3.34 (d, 3H, J = 6 Hz), 3.27 (d, 3H, J = 6.4 Hz), 2.97-2.89 (m, 4H), 2.80 (t, 2H, J = 6.6 Hz), 2.66 (m, 2H), 2.23 (m, 2H), 2.03 (t, 2H, J = 6.8 Hz), 1.34 (m, 2H), 0.76 (m, 3H). ^{13}C{^{1}H} NMR (C_2D_6OS, 100 MHz) δ: 168.3, 164.9, 160.6, 156.4, 153.8, 152.2, 143.8, 136.0, 134.3, 133.1, 129.2, 127.3, 124.6, 123.7, 113.2, 109.2, 102.4, 67.5, 48.6, 45.3, 39.5, 37.6, 37.0, 29.3, 26.6, 25.9, 25.2, 23.7, 21.9. HRMS (ESI-TOF) m/z: $[M + H]^+$ Calcd for $C_{60}H_{53}N_6O_{14}S_2$ 1145.3061; Found 1145.3085. (Figures S11-S13)

asym5-6FAM. The method of **asym5-5FAM** synthesis, which is described above, was followed in order to synthesize the chemical probe **asym5-6FAM**. Here, ***exo*-BCN-9** (41.0 mg, 0.060 mmol, 1 equiv) and **14b** (30.2 mg, 0.066 mmol, 1.1 equiv) were added in sequence to 1,4-dioxane (2 mL), reacted, and worked up to yield

asym5-6FAM (53.8 mg, 0.047 mmol, 78%; Scheme S2) as an orange solid. ^1H NMR (C_2D_6OS, 400 MHz) δ: 9.02 (t, 1H, *J* = 5.2 Hz), 8.72 (t, 1H, *J* = 5.4 Hz), 8.47 (s, 1H), 8.22 (d, 1H, *J* = 9.2 Hz), 8.14 (d, 1H, *J* = 9.2 Hz), 8.07 (d, 1H, *J* = 8 Hz), 7.66 (s, 1H), 7.37 (d, 1H, *J* = 8 Hz), 7.30 (t, 1H, *J* = 5.6 Hz), 6.67 (s, 4H), 6.61-6.52 (m, 8H), 4.22 (t, 2H, *J* = 7 Hz), 3.84-3.73 (m, 2H), 3.61 (q, 2H, *J* = 6.4 Hz), 3.28 (q, 3H, *J* = 6.4 Hz), 3.22 (q, 3H, *J* = 6 Hz), 2.95 (t, 2H, *J* = 6.8 Hz), 2.81 (t, 5H, *J* = 6.6 Hz), 2.67-2.50 (m, 2H), 2.19 (m, 2H), 1.91 (t, 2H, *J* = 7 Hz), 1.28 (m, 2H), 0.75 (m, 3H). $^{13}C\{^1H\}$ NMR (C_2D_6OS, 100 MHz) δ: 168.2, 164.8, 160.7, 156.4, 152.2, 143.7, 140.1, 135.9, 134.2, 133.0, 129.3, 125.2, 124.7, 123.7, 122.7, 113.3, 109.3, 102.3, 67.5, 48.6, 45.1, 39.5, 37.6, 36.9, 29.2, 26.6, 25.8, 25.2, 23.6, 21.8. HRMS (ESI-TOF) m/z: $[M + H]^+$ Calcd for $C_{60}H_{53}N_6O_{14}S_2$ 1145.3061; Found 1145.3024. (Figures S14-S16)

asym6-5FAM. Synthesis of the chemical probe **asym6-5FAM** was similarly performed according to the **asym5-5FAM** synthesis method. A reaction mixture for the **asym6-5FAM** synthesis was prepared by the dispersion of ***exo*-BCN-10** (42.0 mg, 0.061 mmol, 1 equiv) and **14a** (30.9 mg, 0.067 mmol, 1.1 equiv) in sequence into 1,4-dioxane (2 mL). The reaction was allowed to proceed as for **asym5-5FAM**, and the products were purified to afford **asym6-5FAM** (35.7 mg, 0.031 mmol, 51%; Scheme S2) as an orange solid. ^1H NMR (C_2D_6OS, 400 MHz) δ: 8.87 (t, 2H, *J* = 4.8 Hz), 8.46 (s, 1H), 8.21 (d, 1H, *J* = 6.8 Hz), 8.16 (d, 1H, *J* = 6.8 Hz), 8.07 (d, 1H, *J* = 8.4 Hz), 7.68 (s, 1H), 7.37 (d, 1H, *J* = 8 Hz), 7.26 (t, 1H, *J* = 5.4 Hz), 6.67 (s, 4H), 6.61-6.52 (m, 8H), 4.32 (t, 2H, *J* = 7 Hz), 3.77 (t, 2H, *J* = 6 Hz), 3.50 (q, 2H, *J* = 6.4 Hz), 3.35 (q, 3H, *J* = 6.4 Hz), 3.25 (q, 3H, *J* = 6.4 Hz), 2.86 (t, 4H, *J* = 6.8 Hz), 2.74 (t, 2H, *J* = 6.8 Hz), 2.70 (m, 2H), 2.39-2.20 (m, 2H), 2.03 (t, 2H, *J* = 6.8 Hz), 1.31 (m, 2H), 0.75 (m, 3H). $^{13}C\{^1H\}$ NMR (C_2D_6OS, 100 MHz) δ: 168.2, 164.7, 160.7, 156.3, 152.1, 143.8, 139.9, 136.1, 134.2, 133.1, 129.2, 127.3, 125.3, 124.6, 123.6, 122.7,

113.3, 109.3, 102.3, 67.4, 45.3, 39.5, 37.5, 37.0, 36.7, 29.3, 26.6, 25.9, 25.2, 23.6, 21.8. HRMS (ESI-TOF) m/z: $[M + H]^+$ Calcd for $C_{60}H_{53}N_6O_{14}S_2$ 1145.3061; Found 1145.3037. (Figures S17-S19)

asym6-6FAM. The reaction mixture of ***exo*-BCN-10** (45.2 mg, 0.066 mmol, 1 equiv) and **14b** (33.0 mg, 0.072 mmol, 1.1 equiv) in 1,4-dioxane (2 mL) was similarly reacted and worked up according to the **asym5-5FAM** synthesis method to afford the chemical probe **asym6-6FAM** (46.0 mg, 0.040 mmol, 61%; Scheme S2) as an orange solid. ^{1}H NMR (C_2D_6OS, 600 MHz) δ: 8.89 (t, 1H, J = 5.4 Hz), 8.76 (t, 1H, J = 7.2 Hz), 8.15 (t, 2H, J = 4.8 Hz), 8.07 (t, 2H, J = 5.2 Hz), 7.66 (d, 2H, J = 4.8 Hz), 7.27 (t, 1H, J = 4.8 Hz), 6.60 (m, 8H), 6.51 (s, 4H), 4.22 (t, 2H, J = 4.6 Hz), 3.77 (t, 2H, J = 4.6 Hz), 3.48 (q, 2H, J = 4.6 Hz), 3.35 (q, 3H, J = 6.4 Hz), 3.23 (q, 3H, J = 6.4 Hz), 2.84 (t, 4H, J = 5.2 Hz), 2.74 (t, 2H, J = 4.6 Hz), 2.65-2.53 (m, 2H), 2.17 (m, 2H), 1.92 (t, 2H, J = 4.6 Hz), 1.24 (m, 2H), 0.74 (m, 3H). ^{13}C{^{1}H} NMR (C_2D_6OS, 150 MHz) δ: 168.2, 164.7, 160.7, 156.32, 152.65, 143.7, 139.6, 136.0, 134.3, 133.0, 131.4, 129.3, 128.7, 125.6, 124.6, 123.1, 114.6, 109.5, 102.4, 67.4, 45.1, 39.5, 37.5, 36.9, 36.7, 29.2, 26.6, 25.8, 25.1, 23.6, 21.8. HRMS (ESI-TOF) m/z: $[M + H]^+$ Calcd for $C_{60}H_{53}N_6O_{14}S_2$ 1145.3061; Found 1145.2977. (Figures S20-S22)

Spectroscopic measurements. An Amersham Biosciences Ultrospec 2100 pro spectrophotometer (KMU) was used to record UV-Vis absorption spectra and to perform kinetic studies. Fluorescence spectra and kinetic analyses were obtained with a PerkinElmer LS-55 fluorescence spectrometer (KMU) under an excitation wavelength of 325 nm for 6-FAM detection in a 1-cm standard quartz cuvette. As indicated previously,[5] it was necessary to use 325 nm as the excitation wavelength for the FAM derivatives because 475 nm excitation light – the standard wavelength for

FAM excitation – interfered with the measurements of the 6-FAM fluorescence emission at 515 nm in the fluorescence spectrometer.

Fluorescence quantum yield measurements. Values of absolute fluorescence quantum yield (Φ_A) for **11, asym5-5FAM, asym5-6FAM, asym6-5FAM, and asym6-6FAM** were determined using an FS5 Spectrofluorometer (Edinburgh Instruments, Livingston, United Kingdom) equipped with an SC-30 150 mm diameter integrating sphere module (KMU) and were calculated by following instructions of the manufacturer. The excitation wavelength was 470 nm in this study.

Specificity, reactivity, kinetics and mechanism of reactions between the fluorescent chemical probe asym6-6FAM and thiols. The specificity and reactivity of **asym6-6FAM** were characterized by obtaining pseudo-first-order rate constant (k_1) values. In a typical experiment for k_1 determination, a quartz cuvette containing 1.341 mL of phosphate buffer (PB; 100 mM, pH 7.4) was first mounted onto the temperature controller of the PerkinElmer LS-55 fluorescence spectrometer. The measurement was performed after sequential transfer of 150 µL of a 50 mM solution of one of the reactants [DL-dithiothreitol (DTT), L-glutamate, glycine, L-cysteine, glutathione (GSH), L-serine, L-lysine, L-methionine, 2-mercaptoethanol (2-ThioEtOH), 2-aminoethanethiol (2-AET), thiocholine or 1-butanethiol (nBuSH)] in PB and 9 µL of **asym6-6FAM** (25 µM in DMSO) to the PB solution in the cuvette to give [reactant] = 5 mM and [**asym6-6FAM**] = 0.15 µM in the reaction. Progress of the reaction at 25°C was followed by measuring 6-FAM fluorescence (λ_{max} = 515 nm) until signal saturation was observed, typically after 15-110 min. Normalized fluorescence intensity at 515 nm was calculated by subtracting the background fluorescence intensity of **asym6-6FAM** at 515 nm from the original measurement of

fluorescence intensity at 515 nm. The normalized data were fitted to a single-exponential equation for first-order kinetics $F(t) = F_0 + F_{max}(1 - e^{-k_1 t})$ [$F(t)$, normalized 6-FAM fluorescence at a specific time point t] to give k_1 (GraphPad, La Jolla, CA, USA). Analogous pseudo-first-order kinetic analysis was employed to determine a second-order rate constant (k_2) in which [2-AET] in the **asym6-6FAM** (0.5 μM)-containing reaction was 1 mM, 2.5 mM, 5 mM, 7.5 mM, 10 mM, 12.5 mM, or 15 mM. A k_2 value was calculated according to the slope of the linear regression from the plot of k_1 *vs* [2-AET]. pH effects on the reaction of **asym6-6FAM** (0.5 μM) with 2-AET (5 mM) were determined by the same pseudo-first-order kinetics in the presence of the following four buffers and pH ranges: MES (pH 5.5-6.5), PIPES (pH 6.5-7.4), EPPS (pH 7.4-8.6), and CHES (pH 8.6-9.5). The pH titration study provided data for calculating the pK_{a1} value for 2-AET according to the equation $k_1 = k_{1,max}[1 + 10^{(pK_{a1} - pH)}]$ (GraphPad, USA). Similarly, the 2-AET (5 mM)**-asym6-6FAM** (0.15 μM) reactions in the presence of 1 mM of metal ions [K^+, Li^+, Na^+, Ca^{2+}, Cd^{2+}, Co^{2+}, Cu^{2+}, Mg^{2+}, Mn^{2+}, Ni^{2+}, Zn^{2+}, Fe^{3+}] were used to determine the corresponding k_1 values in order to understand the effects of metal ions on the original 2-AET**- asym6-6FAM** reaction. Kinetic experiments were performed a minimum of three times for each reaction; the provided k_1 values were the mean ± SD of the experiments and were used to plot Figure S3 in SI.

The chemical probe asym6-6FAM for developing fluorescence turn-on assays and for measuring the activities of butyrylcholinesterase (BChE) and PON1 lactonase. Activity of BChE from equine serum (Merck Ltd., Taiwan) was expressed as U L^{-1} in which one unit (1 U) of BChE activity was defined as the amount of BChE able to hydrolyze 1 μmol of *S*-butyrylthiocholine iodide (BTCh) per min. In addition, the recombinant PON1 (rePON1), which was shown to have

catalytic activity and function similar to human PON1,[10] was overexpressed and subsequently purified according to an established method.[4, 5] Purified rePON1 protein concentration was determined by the Bradford assay (Bio-Rad). PON1 lactonase activity was equivalent to rePON1 lactonase activity and was expressed as U L^{-1} in which one unit (1 U) was similarly defined as the amount of PON1 able to hydrolyze 1 μmol of TBBL per min.[8] Activity of BChE and PON1 lactonase was calibrated using Ellman's colorimetric method.[4, 7]

In the **asym6-6FAM**-based fluorescence turn-on assay for quantitative measurements of BChE activity, a solution of **asym6-6FAM** (0.3 μM, 0.6% DMSO), BChE (1.9–182.2 U L^{-1}), and BTCh (1 mM) in PB was prepared in a quartz cuvette previously mounted onto the temperature controller of the PerkinElmer LS-55 fluorescence spectrometer. BChE catalysis was measured at 37 °C for 15 min. Reaction progression was monitored by the fluorescence intensity at 515 nm as a function of reaction time. The normalized fluorescence intensity at 515 nm was acquired by subtracting a background fluorescence intensity at 515 nm of **asym6-6FAM** from the original reading of fluorescence intensity at the same wavelength. The fluorescence changes of the steady-state reactions were employed to calculate the initial rate (v_i) of BChE catalysis. Each specific BChE catalysis reaction was performed a minimum three times with the reported v_i as the mean ± SD of the experiments.

PON1 lactonase activity was determined by utilizing the **asym6-6FAM**-based assay in solutions containing **asym6-6FAM** (0.7 μM in DMSO, 1.4%), rePON1 (15.8-316.1 U L^{-1}, 0.17-3.33% glycerol), and TBBL [10 mM in acetonitrile, 1%] in Tris buffer (50 mM Tris, 1 mM Ca^{2+}, pH 8.0) using the quartz cuvette previously mounted onto the temperature controller of the PerkinElmer LS-55 fluorescence spectrometer. PON1 catalysis proceeded at 25˚C for 30 min; reaction progression was

monitored by measuring the fluorescence intensity at 515 nm as a function of reaction time. The normalized fluorescence intensity data at 515 nm were attained by subtracting background fluorescence of **asym6-6FAM** at the same wavelength and same reaction time from the original fluorescence intensity reading. Initial rates (v_i) of PON1 lactonase catalysis were also determined from the fluorescence changes of the steady state reactions. Each specific PON1 catalysis reaction was repeated a minimum of three times with the reported v_i as the mean ± SD of the experiments.

Activities of BChE and PON1 lactonase in human serum and effects of an inhibitor on BChE catalysis. Activity of human BChE and PON1 lactonase was measured after a trained phlebotomist at KMU Hospital drew 20 ml of whole blood from three healthy male volunteers. The blood samples were centrifuged, and the serum was separated from the plasma. A 100-fold dilution of the serum was used as a substitute for BChE in the **asym6-6FAM**-based assay. Human PON1 lactonase activity was quantified by employing a serum aliquot diluted 750-fold as a substitute for rePON1 in separate **asym6-6FAM**-based reactions.

It is noted that, before adding BTCh (1 mM in PB) for quantifying BChE activity or before adding TBBL (10 mM in ACN, 1%) for measuring PON1 lactonase activity, GSH and other biothiols in diluted serum was consumed in the presence of **asym6-6FAM** (0.3 μM in DMSO, 0.6%) in PB at 37 °C for 1 h or **asym6-6FAM** (0.7 μM in DMSO, 1.4%) in Tris buffer at 25°C for 1 h, respectively.

The potential for the fluorescence turn-on assay based on the fluorogenic properties of **asym6-6FAM** to be used in measuring the inhibition of BChE catalysis and in developing platforms for screening BChE inhibitors useful in drug discovery was confirmed by studying the effects of the inhibitor tacrine on BChE catalysis. Each reaction contained **asym6-6FAM** (0.3 μM, 0.6% DMSO), BChE (136.66 U L^{-1}),

BTCh (250 or 300 μM), and tacrine (50, 100, or 200 nM) in PB. Each BChE catalysis reaction in the presence of tacrine was studied by the **asym6-6FAM**-based assay in the PerkinElmer LS-55 fluorescence spectrometer and performed three times with the reported v_i as the mean ± SD. The inhibitor constant (K_i) value of tacrine on BChE catalysis was determined from a plot of [tacrine] *vs* v_i^{-1} by taking the absolute value of the *x*-axis value at the point of intersection of the two lines in the plot. Each line represented a series of BChE catalysis reactions in the presence of tacrine and a [BTCh] of either 250 or 300 μM.

High-throughput asym6-6FAM-based fluorescence turn-on assay for measuring human BChE activity in serum. The chemical probe **asym6-6FAM** was exploited to develop a high-throughput fluorescence turn-on assay for determining BChE activity by preparing a solution of **asym6-6FAM** (9 μM, 1.8% DMSO), BChE (10–182.2 U L^{-1}), and BTCh (1 mM) in PB (100 mM, pH 7.4). The solutions were transferred to individual wells of a black, flat-bottomed, 96-well, chimney microplate (Greiner, Kremsmünster, Austria). The final solution volume in each well was 0.2 mL. The plate was immediately placed in a BioTek Synergy HTX multimode microplate reader (KMU). BChE catalysis was measured at 37 °C for 30 min while the plate was shaken every 30 s. The 6-FAM fluorescence was read with a 485/20 nm filter and a 528/20 nm filter. The normalized 6-FAM fluorescence was acquired by subtracting background readings, which were determined from a well containing **asym6-6FAM** and BTCh in the absence of BChE in PB, from the original readings of 6-FAM fluorescence in samples. The fluorescence changes of the steady-state reactions were employed to calculate v_i of BChE catalysis. A plot of BChE activity *vs* v_i was obtained to establish a calibration equation and to determine BChE activity of unknowns.

For quantifying BChE activity in human sera, a 100-fold dilution of each serum sample was used to substitute for BChE in the high-throughput **asym6-6FAM**-based assay. Similarly, the fluorescence changes of the steady-state reactions in serum samples were employed to calculate v_i of BChE catalysis and to determine BChE activity according to the calibration equation mentioned above. Each specific BChE catalysis reaction was performed in triplicate with the reported v_i as the mean ± SD of the experiments.

References

(1) Rady, T.; Mosser, M.; Nothisen, M.; Erb, S.; Dovgan, I.; Cianférani, S.; Wagner, A.; Chaubet, G. Bicyclo[6.1.0]nonyne carboxylic acid for the production of stable molecular probes. *RSC Adv.* **2021**, *11*, 36777-36780.
(2) Dommerholt, J.; Schmidt, S.; Temming, R.; Hendriks, L. J. A.; Rutjes, F. P. J. T.; van Hest, J. C. M.; Lefeber, D. J.; Friedl, P.; van Delft, F. L. Readily accessible bicyclononynes for bioorthogonal labeling and three-dimensional imaging of living cells. *Angew. Chem., Int. Ed.* **2010**, *49*, 9422-9425.
(3) Gong, M.-M.; Dai, C.-Y.; Severance, S.; Hwang, C.-C.; Fang, B.-K.; Lin, H.-B.; Huang, C.-H.; Ong, C.-W.; Wang, J.-J.; Lee, P.-L.; Wang, T.-P. A Bioorthogonally synthesized and disulfide-containing fluorescence turn-on chemical probe for measurements of butyrylcholinesterase activity and inhibition in the presence of physiological glutathione. *Catalysts* **2020**, *10*, 1169.
(4) Fang, B.-K.; Dai, C. Y.; Severance, S.; Hwang, C.-C.; Huang, C.-H.; Hou, S.-Y.; Yeh, B.-L.; Gong, M.-M.; Chou, Y.-H.; Wang, J.-J.; Wang, T.-P. Sensitive assay for the lactonase activity of serum paraoxonase 1 (PON1) by harnessing the fluorescence turn-on characteristics of bioorthogonally synthesized and geometrically controlled chemical probes. *Molecules* **2022**, *27*, 2435.
(5) Huang, C.-H.; Hou, S.-Y.; Severance, S.; Hwang, C.-C.; Fang, B.-K.; Gong, M.-M.; Yu, S.-L.; Weng, Y.-C.; Wang, L.-F.; Dai, C.-Y.; et al. Manipulating diastereomeric bicyclononynes to sensitively determine enzyme activity and facilitate macromolecule conjugations. *ACS Omega* **2023**, *8*, 46073-46090.
(6) Lee, M.; Grissom, C. B. Design, Synthesis, and characterization of fluorescent cobalamin analogues with high quantum efficiencies. *Org. Lett.* **2009**, *11*, 2499-2502.
(7) Ellman, G. L.; Courtney, K. D.; Andres, V.; Featherstone, R. M. A new and rapid colorimetric determination of acetylcholinesterase activity. *Biochem. Pharmacol.* **1961**, *7*, 88-95.
(8) Khersonsky, O.; Tawfik, D. S. Chromogenic and fluorogenic assays for the lactonase activity of serum paraoxonases. *ChemBioChem* **2006**, *7*, 49-53.
(9) Bernardes, G. J. L.; Chalker, J. M.; Errey, J. C.; Davis, B. G. Facile conversion of cysteine and alkyl cysteines to dehydroalanine on protein surfaces: versatile and switchable access to functionalized proteins. *J. Am. Chem. Soc.* **2008**, *130*, 5052-5053.
(10) Aharoni, A.; Gaidukov, L.; Yagur, S.; Toker, L.; Silman, I.; Tawfik, D. S. Directed evolution of mammalian paraoxonases PON1 and PON3 for bacterial expression and catalytic specialization. *Proc. Natl. Acad. Sci. U.S.A.* **2004**, *101*, 482-487.

Glossary

AChE, acetylcholinesterase; 2-AET, 2-aminoethanethiol; BChE, butyrylcholinesterase; BTCh, *S*-butyrylthiocholine iodide; DTT, DL-dithiothreitol; *exo*-BCN, *exo*-bicyclo[6.1.0]nonyne; FAM, carboxyfluorescein; 5-FAM, 5-carboxyfluorescein; 6-FAM, 6-carboxyfluorescein; FRET, Förster resonance energy transfer, GSH, glutathione; 2-HQ, 2-hydroxyquinoline; k_1, pseudo-first-order rate constant; k_2, second-order rate constant; LOD, limit of detection; nBuSH, 1-butanethiol; PB, phosphate buffer; PeT, photoinduced electron transfer; PON1, paraoxonase 1; rePON1, recombinant PON1; SPAAC, strain-promoted azide-alkyne cycloaddition; TBBL, 5-thiobutyl butyrolactone; TBET, through-bond energy transfer; 2-ThioEtOH, 2-mercaptoethanol; v_i, initial rate.
